# On separating long- and short-term memories in hyperdimensional computing

**DOI:** 10.3389/fnins.2022.867568

**Published:** 2023-01-09

**Authors:** Jeffrey L. Teeters, Denis Kleyko, Pentti Kanerva, Bruno A. Olshausen

**Affiliations:** ^1^Redwood Center for Theoretical Neuroscience, University of California, Berkeley, Berkeley, CA, United States; ^2^Intelligent Systems Lab, Research Institutes of Sweden, Kista, Sweden

**Keywords:** hyperdimensional computing, vector symbolic architectures, sparse distributed memory, long-term memory, holographic reduced representation, associative memory, short-term memory, working memory

## Abstract

Operations on high-dimensional, fixed-width vectors can be used to distribute information from several vectors over a single vector of the same width. For example, a set of key-value pairs can be encoded into a single vector with multiplication and addition of the corresponding key and value vectors: the keys are bound to their values with component-wise multiplication, and the key-value pairs are combined into a single superposition vector with component-wise addition. The superposition vector is, thus, a memory which can then be queried for the value of any of the keys, but the result of the query is approximate. The exact vector is retrieved from a codebook (a.k.a. item memory), which contains vectors defined in the system. To perform these operations, the item memory vectors and the superposition vector must be the same width. Increasing the capacity of the memory requires increasing the width of the superposition and item memory vectors. In this article, we demonstrate that in a regime where many (e.g., 1,000 or more) key-value pairs are stored, an associative memory which maps key vectors to value vectors requires less memory and less computing to obtain the same reliability of storage as a superposition vector. These advantages are obtained because the number of storage locations in an associate memory can be increased without increasing the width of the vectors in the item memory. An associative memory would not replace a superposition vector as a medium of storage, but could augment it, because data recalled from an associative memory could be used in algorithms that use a superposition vector. This would be analogous to how human working memory (which stores about seven items) uses information recalled from long-term memory (which is much larger than the working memory). We demonstrate the advantages of an associative memory experimentally using the storage of large finite-state automata, which could model the storage and recall of state-dependent behavior by brains.

## 1. Introduction

Hyperdimensional (HD) computing, also known as Vector Symbolic Architectures (Gayler, 2003; Kanerva, 2009; Kleyko et al., 2022c,d), with origins in Holographic Reduced Representation (Plate, 1994a, 1995), is an approach to perform computations using vectors that contain many (in order of at least hundreds) of components. In HD computing, each basic concept within a domain is associated with a single vector. A measure of similarity between vectors is specified, for example, the Hamming distance if binary vectors are used. The computations to be performed, such as formation of representation of compositional concepts, are implemented using operations on, and between, the vectors. Commonly, three operations are defined:

- Addition (denoted as +), also called “bundling” or “superposition,” which takes several vectors and produces a vector similar to all of the input vectors.
- Multiplication (denoted as ○), also called “binding,” which takes two vectors and generates a vector dissimilar to both input vectors. In the case of binary and bipolar vectors (which were used in this study), the binding operation can be reversed (“unbound” or “released”) by multiplying the result of binding by one of the input vectors to retrieve the other input vector. The binding operation distributes over addition.
- A permutation operation—denoted as *ρ*()—which takes one input vector and produces a vector dissimilar to it. The permutation operation distributes over both addition and multiplications, and it has an inverse.

A fundamental decision that must be made when designing a system with HD computing is how wide should the vectors be (that is, how many components should they contain)? The vectors must be wide enough to allow the functionality (e.g., retrieval from the distributed vector representations) to be performed with the desired accuracy. However, if the vectors are too wide, then the system will be less efficient because storage space or computations or both will be used inefficiently.

Of the three operations used in HD computing mentioned above, the addition operation is the one that mainly determines how wide the vectors must be. This is because a vector generated using the addition operation (called a superposition vector) is usually compared to other vectors stored in a codebook (using a measure of similarity) to find those that were used to form the superposition. The more vectors that are added when forming a superposition vector, the wider the vectors must be to allow high retrieval accuracy. This phenomenon was investigated in, e.g., Plate (1994a), Gallant and Okaywe (2013), and Thomas et al. (2021) and treated in great details by Frady et al. (2018).

A data structure primitive that is often performed in HD computing that makes extensive use of the addition operation is to use a superposition vector as a memory for a set of key-value pairs (Kanerva, 2009; Kleyko et al., 2022a). To store key-value pairs in a superposition vector, the binding and addition operations are used together. For example, if key-value pairs are: (**k**_1_, **v**_1_) and (**k**_2_, **v**_2_) these can be encoded into a superposition vector (**s**) by setting **s** = (**k**_1_ ○ **v**_1_) + (**k**_2_ ○ **v**_2_). The releasing operation can be performed by multiplying **s** with the inverse of a key, which is the key itself for binary and bipolar vectors. For example **k**_1_ ○ **s**, to return a vector with a high similarity to the matching value (**v**_1_). Sequences can also be stored in a superposition vector using the same method, with each key set to vector that is generated from the position of the item in the sequence. For instance, the key can be formed by permuting a particular vector by the number of times corresponding to the position (Plate, 1992; Kanerva, 2009).

To enable storage of key-value pairs in this way (using a superposition vector), the system must have a memory containing the possible vectors that are read out in order to find the one most similar to the result of releasing. This memory is called the “item memory,” “cleanup memory,” or “codebook.”

Because a superposition vector is formed by the addition operation, a characteristic of systems that store information in a superposition vector is that the minimum width of the superposition vector is inextricably linked to the number of items that the system can store with a given level of reliability. That is, the more items that must be stored, the wider the superposition vector must be. Another characteristic is that since representations are of fixed width, the width of the superposition vector determines the width of all other vectors including those in the item memory. Thus, even for moderately sized item memories, if the item memory is explicitly represented, most of the storage required to implement the system will be used by the item memory (but in certain scenarios hashing might help, see Thomas et al., 2022).

Nevertheless, if only a small number of items are to be stored in the superposition vector, these two characteristics could have a minimal effect on the total storage required for the system because all the vectors must be a minimal width to facilitate HD computing and the increase in vector width needed for storing items in the superposition vector will be relatively small. However, if there are many items that should be stored, the additional width required for the superposition vector to allow reliable storage will increase greatly the total storage required to implement the system.

At a high level of abstraction, one can think about the examples above in terms of using the superposition vector either as short-term or long-term memory. Thus, we suggest to only consider the superposition vector for short-term memory and look for alternative solutions to implement the long-term memory.

To investigate how the storage of key-values pairs might be stored more efficiently for HD computing, we experimented with using an associative memory (Gritsenko et al., 2017) for vectors and compare that to using a superposition vector. The associative memory that we used in these experiments is the Sparse Distributed Memory (SDM) (Kanerva, 1984, 1988, 1993). With the SDM, the capacity of the memory is not determined by the width of the vectors, but instead, by the number of memory locations that are included in the SDM. This decoupling of vector width from memory capacity allows creating a system which has an arbitrarily large capacity, without requiring the width of vectors in the item memory to increase.

To do the experiments we compared the computational resources (storage space and computations) required to store a large finite-state automaton with the same recall error rate using two variations of the superposition vector memory and four variations of the SDM. Different variations of the superposition vector and SDM were used because each variant requires different computational resources to attain the same recall error rate so considering different variations allows making a more general conclusion regarding the comparison of a superposition vector and an associative memory. Also, some variations (those that threshold—or binarize—the memory, i.e., make into vectors of 0s and 1s, or 1s and −1s, as appropriate) are only suitable if changes to the memory are not needed after the data is stored, while those that do not binarize the memory are suitable for continual learning.

The article is organized as follows: Section 2 describes the superposition vector and associative memories, and the finite-state automata. It also gives equations used to predict the error rate of the memories. Section 3 gives the dimensions of the different types of memories required to store a large finite-state automaton at different error rates, then uses those dimensions to compare the performance of the superposition vector and associative memories with respect to the space (bits) and computations required for recall. Section 4 summarizes the results and describes some of the broader implications.

## 2. Materials and methods

### 2.1. Superposition vector memory

Two types of superposition vector memory were used in the experiments. The first, called here “***S1***,” used a binary vector to store the data, while the second (called “***S2***”) used a vector of 8-bit integer values to store the data. The implementation of ***S1*** is described first, followed by the description of ***S2***. It is worth noting that throughout the article, we consider two well-known models of HD computing: Binary Spatter Codes (Kanerva, 1996) and Multiply-Add-Permute (Gayler, 1998) that use binary and bipolar vectors, respectively. Under some assumptions, these models are interchangeable. We will actively use this fact below when introducing memory models investigated in this study. Note that the family of HD computing includes many other models, for example, those computing with sparse binary vectors (Rachkovskij, 2001; Laiho et al., 2015; Kleyko et al., 2016; Frady et al., 2021b). This topic is, however, outside the scope of this article but we refer interested readers to Schlegel et al. (2022) and Kleyko et al. (2022c) that treat the topic in detail.

#### 2.1.1. Variant *S1*

The implementation of ***S1*** matches the description provided in Kanerva (1997).

- Binary vectors (of width *n*_*s*_) are used for both vectors in the item memory and the superposition vector. The vectors in the item memory are initialized to random 0s and 1s.
- The superposition vector is created as follows: First, an array of integer counters of the same width as the superposition vector is initialized to zeros. The range of each counter is limited to [−127, 127] so each counter can be stored using 8 bits. For each bit in each vector stored in the memory, the counter aligned to the position of the bit is incremented if the bit is one or decremented if the bit is zero. The counters are then binarized to {0, 1}. To do that, first, if an even number of vectors are stored, a random binary vector is included so that the number of stored vectors is odd. Then bits in the binary superposition vector are set to 1 if the sum in the corresponding counters are positive, and to 0 if they are negative. Note that since an odd number of vectors are stored, the counter sums can never be zero.
- A key-value pair is stored in the superposition vector memory by binding the key and value vectors using component-wise XOR and then storing the resulting vector in the memory as described above (incrementing or decrementing the counters).
- The recall of a value associated with a key is accomplished by computing the XOR of the key with the superposition vector. This result is compared to vectors in the item memory using the Hamming distance and the vector which most closely matches is selected as the recalled value. If there are more than one vector that have the same smallest Hamming distance, then either one of them is selected at random, or this could be counted as an error in recall. In this study, the latter was done.

#### 2.1.2. Variant *S2*

The ***S2*** Superposition vector operates similarly to ***S1***, with the following changes:

- Bipolar vectors {−1, +1} are used for the item memory instead of binary vectors.
- Binding between the key and value vectors is done by component-wise multiplication instead of XOR. (As with ***S1***, the result—a bipolar vector—is added to the counters).
- The superposition vector is set to the vector of counters after the data vectors are stored (the counters are not binarized).
- The recall of a value is performed by component-wise multiplication of a key and the superposition vector and the resulting vector is then compared with vectors in the item memory using the dot product; the vector in the item memory which has the largest dot product is selected as the matching value.

A result of these changes is that the superposition vector used for ***S2*** has 8 bits per component, whereas the superposition vector used in ***S1*** has only one bit per component. As will be shown in Section 3, this difference causes the two variations to require different amounts of storage space and computation to have the same reliability. The two variations are summarized in the first two rows of Table 1.

**Table 1:** Variations of superposition vector memory and the SDM.

**Name**	**Binarized memory**	**Threshold sum**
***S1***	Yes	–
***S2***	No	–
***A1***	No	Yes
***A2***	Yes	Yes
***A3***	Yes	No
***A4***	No	No

The superposition vector memories do not sum counters, so the last column is not applicable to those.

### 2.2. Sparse distributed memory

**Figure 1:**
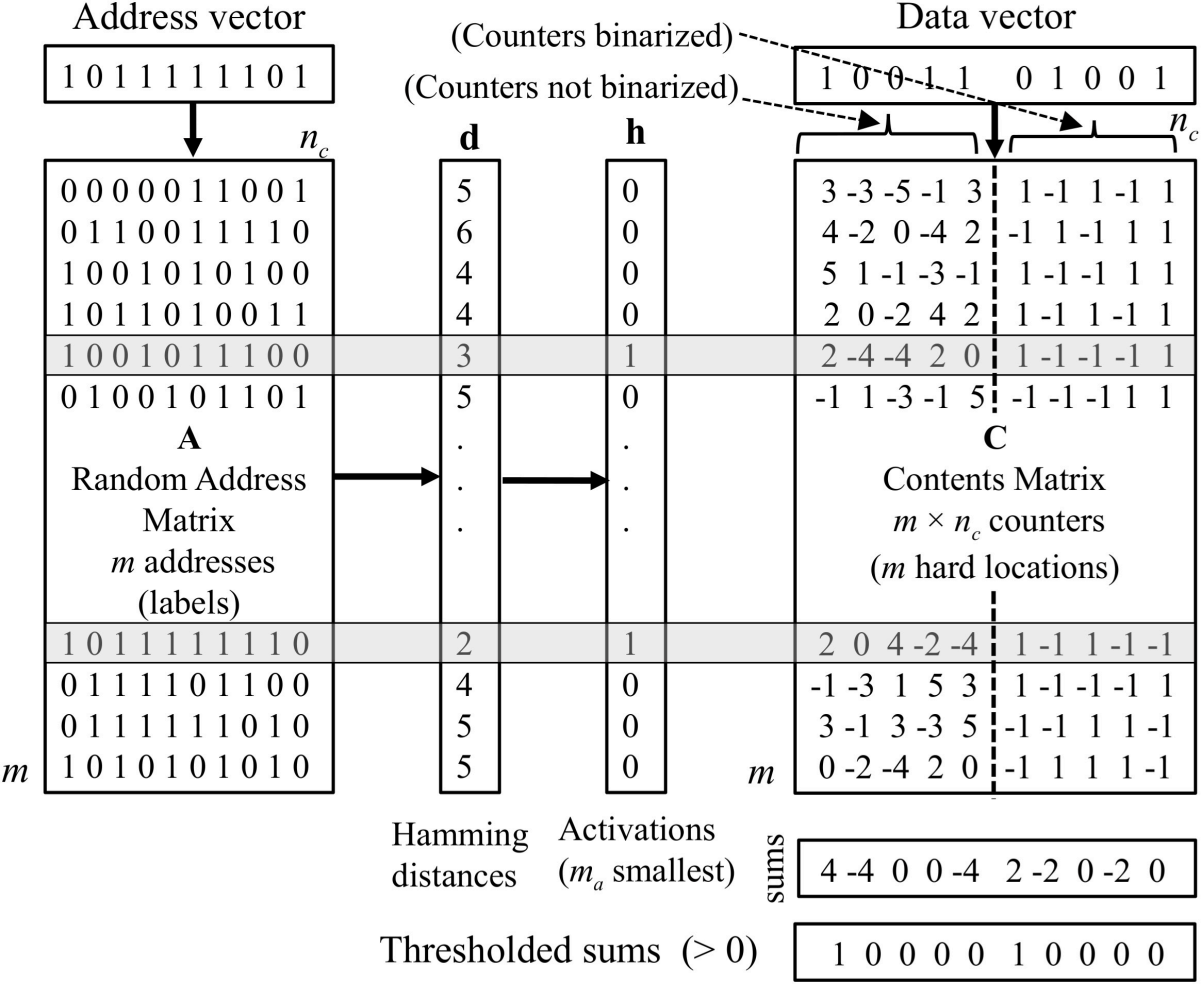
An outline of the organization of the SDM. The memory locations activated for storage or recall phases (shaded rows) are those with the smallest Hamming distance between the address vector and addresses (labels) in the address matrix. To store data, counters in the activated rows (hard locations) in the contents matrix are incremented or decremented according to the bits in the data vector. For variations ***A2*** and ***A3*** the counters are binarized before recall. The recall is done by column-wise superposition (adding) the counters in activated rows and thresholding at zero for variations ***A1*** and ***A2***.

We evaluate four variations of the SDM, which we refer to as “***A1***,” “***A2***,” “***A3***,” and “***A4***.” All of these variations operate the same way during the data storage phase. Unlike the superposition vector memory, which takes single vectors as input (and stores them by adding them together using the addition operation), the SDM takes pairs of vectors as input. Each pair consists of an “address vector” and a “data vector.” Recall is done by presenting an address vector, then recalling the data vector, which should be associated with that address, using the corresponding SDM's algorithm. In essence, the address vector is a key, and the data vector is a value. Figure 1 illustrates the organization of the SDM.

The storage of data in the SDM is similar to the storage of data in a superposition vector in that there are integer counters in the SDM that are initialized to zero and incremented or decremented according to the value of the corresponding bit of the data vector being stored.^1^ However, instead of there being just one row of counters (as is the case with the superposition vector), there is a matrix of counters (the “contents matrix”). Each row in the matrix is a storage location and only a small fraction of them are activated to store and recall the data vector associated with a particular address vector. To determine which rows of counters are activated by an address vector, each row in the memory, referred to as a “hard location,” is associated with a fixed random label that is a row in the “address matrix” **A** (see Figure 1). The hard locations whose labels most closely match the address vector are activated. This selection can be done by choosing those within a certain Hamming distance, or by choosing a fixed number of rows with the smallest Hamming distance. In the experiments reported in this article, the latter method is used.

Storage of the data vector in an address–data pair is accomplished by incrementing or decrementing the counters in all the activated storage locations according to the value of the corresponding bits in the data vector as done with the superposition vector.

Up to this point, all of the SDM variations (***A1***-***A4***) operate in the same manner. The computations performed to read data from the SDM and find the closest matching vector in the item memory are, however, different for each variant so we specify them below.

#### 2.2.1. Variant *A1*

This is the most commonly described variant of the SDM. During the recall phase, the corresponding counters in the activated rows are added together and thresholded at zero, so that each bit in the returned data is one if the corresponding counter sum is greater than zero, and zero otherwise. The search in the item memory is done using the Hamming distance.

#### 2.2.2. Variant *A2*

This variant operates in the same way as ***A1***, but before the recall phase, each counter in the contents matrix is binarized to {−1, +1} by a procedure similar to that used to binarize the counters in variant ***S1*** of the superposition vector (see Section 2.1.1).

#### 2.2.3. Variant *A3*

A binarized contents matrix is used (as in variant ***A2***), however, the sums of the counters are not thresholded, and the search in the item memory is done by finding the vector in the item memory that has the largest dot product with the recalled vector as is done with variant ***S2*** of the superposition vector (see Section 2.1.2).

#### 2.2.4. Variant *A4*

The contents matrix counters are not binarized and the sums are not thresholded. As with variant ***A3***, the search in item memory is done using the dot product.

#### 2.2.5. Comparison of SDM variations

For both ***A3*** and ***A4***, components of the vectors in item memory are bipolar to enable the dot product to be used to find the closest match to the non-thresholded recalled vector, and components of the address matrix are also bipolar to allow calculating the Hamming distance between location labels and bipolar vectors. An overview of the SDM operation is given in Figure 1. The different variations of the SDM are summarized in Table 1.

In our experiments, the number of hard locations activated by each address vector used for storing or recalling data is set to:


(1) $$\begin{array}{l} m_{a}=\left\lfloor m/\left({\left(2\;k\;m\right)}^{\left(1/3\right)}\right)\right\rceil , \end{array}$$

where *m* is the number of locations (rows) in the SDM and *k* is the number of items (data vectors) to be stored in the SDM and ⌊·⌉ indicates rounding to the nearest positive integer for variations ***A1***, ***A3*** and ***A4***; and to the nearest odd integer for variant ***A2***. This value is used because it was shown to be optimal for variant ***A1*** (Kanerva, 1988, 1993).^2^ An odd value for *m*_*a*_ is used for variant ***A2*** since with this variant the recalled vector is the superposition of *m*_*a*_ bipolar vectors. Thus, an odd *m*_*a*_ is useful for preventing zeros in the recalled vector before thresholding because a zero provides no information about the recalled bit value. To select the *m*_*a*_ hard locations for an address vector, the Hamming distance to all of the hard location labels is found and the locations corresponding to the *m*_*a*_ closest matches are activated. If there are multiple sets of locations that have the closest matches, one of them is selected deterministically so that the same address always activates the same set of *m*_*a*_ locations. For the results given in Section 3, the Python NumPy argpartition function with introselect as the selection algorithm was used to select the *m*_*a*_ closest matches.

For all variations, the width of the vectors (*n*_*c*_) was set to 512 because that is wide enough to ensure clean separability when matching vectors to the item memory, but not so wide that the item memory would take up too much space.

### 2.3. Finite-state automata

HD computing has been used in various application domains. Some examples are classification (Rahimi et al., 2019; Diao et al., 2021; Osipov et al., 2022), clustering (Bandaragoda et al., 2019; Imani et al., 2019), communications (Jakimovski et al., 2012; Kleyko et al., 2012; Kim, 2018), cognitive architectures (Plate, 1994b; Rachkovskij, 2004; Eliasmith, 2013), and approximation of kernel-based methods (Frady et al., 2021a, 2022). In the scope of this article, we focus on studying various memory variations rather than on a particular application scenario. Therefore, to compare the performance of the different memory variations, we store in each system a large finite-state automaton that has been shown before to be represented via HD computing. In this section, we describe what finite-state automata are and how they can be stored using a superposition vector and SDM.

**Figure 2:**
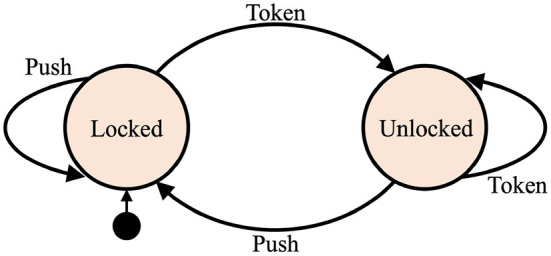
An example of a state diagram of a finite-state automaton modeling the control logic of a turnstile. It has two states and two possible inputs. The start state is depicted by the arrow pointing from the black circle.

A deterministic finite-state automaton is an abstract computational model that is specified via a finite set of allowed input symbols, a finite set of states, a transition function, the start state, and a finite set of accepting states. The automaton is always in one of its possible states. The current state can change in response to an input. The current state and input symbol together uniquely determine the next state of the automaton. Changing from one state to another is called a transition. All possible transitions in the automaton are defined by the transition function. To illustrate the concept of finite-state automaton, an intuitive example of controlling logic of a turnstile is presented in Figure 2. The set of states is {“Locked”, “Unlocked”} and the set of input symbols is {“Push”, “Token”} and the transition function can be easily derived from the state diagram.

HD computing-based implementations of finite-state automata were proposed in Osipov et al. (2017) and Yerxa et al. (2018). The transformation involves all three HD computing operations and uses two item memories. One item memory stores random vectors corresponding to the set of states (denoted as **l** for “Locked” and **u** for “Unlocked”). Another item memory stores random vectors corresponding to the set of input symbols (denoted as **p** for “Push” and **t** for “Token”). The vectors from the item memories are used to form a vector which represents the transition. For example, the transition from “Locked” state to “Unlocked” state, contingent on receiving “Token,” is represented as:


$$\begin{array}{l} \text{t}○\text{l}○\rho \left(\text{u}\right). \end{array}$$

#### 2.3.1. Storing a finite-state automaton using a superposition vector

Given the vector representations of all transitions of the automaton, the transition function of the automaton is represented by the superposition (denoted as **s**) of the individual transitions:


(2) $$\begin{array}{l} \text{s}=\text{p}○\text{l}○\rho \left(\text{l}\right)+\text{t}○\text{l}○\rho \left(\text{u}\right)+\text{p}○\text{u}○\rho \left(\text{l}\right)+\text{t}○\text{u}○\rho \left(\text{u}\right). \end{array}$$

Depending on the variant of the superposition vector memory, **s** can be either binarized (variant ***S1***) or kept as is (variant ***S2***). In order to execute the automaton, we need to query the vector of the transition function for the next state given the current state and input. Therefore, we query **s** with the binding of the vectors of the current state and input symbol followed by the inverse permutation operation applied to the result. Calculated in this way, the result is the noisy version of the vector representing the next state. For example, if the current state is **l** and the input symbol is **p** then we have:


$$\begin{array}{l} \rho^{-1}\left(\text{s}○\text{p}○\text{l}\right)=\text{l}+\text{noise}. \end{array}$$

Finally, this vector should be passed to the item memory in order to retrieve the noiseless vector **l**.

This example can be generalized as follows: Each transition to be stored consists of an initial state (**s**_*i*_), an input (**p**_*j*_), and a next state (**s**_*k*_). To store each transition in the superposition vector **s**, a vector formed by **s**_*i*_ ○ **p**_*j*_ ○ *ρ*(**s**_*k*_) is added to the superposition vector **s**. To recall from the superposition vector given a state **s**_*i*_ and input **p**_*j*_, the recalled (noisy) next state (**r**) is obtained by: $\text{r}=\rho^{-1}\left(\text{s}○{\text{s}}_{i}○{\text{p}}_{j}\right)$. The recalled vector **r** must then be used to retrieve the noiseless next state (**s**_*k*_) from the item memory.

#### 2.3.2. Storing a finite-state automaton using a SDM

To store each transition in the SDM, the address vector (**a**_*ij*_) is set to **a**_*ij*_ = **s**_*i*_ ○ **p**_*j*_ and the data vector is set to **a**_*ij*_ ○ *ρ*(**s**_*k*_). During the recall phase, given a state and input vector, the address vector is formed as above. Once the data vector (denoted as **d**) is recalled from the SDM, the noisy next state is computed by $\text{r}=\rho^{-1}\left({\text{a}}_{ij}○\text{d}\right)$. The reason the address vector is bound to form the data vector before storing is to reduce interference between the same next state being stored from different transitions in the SDM contents matrix. Finally, the noisy vector **r** of the next state is then used to select the noiseless next state (**s**_*k*_) using the item memory.

#### 2.3.3. Interference between terms

In this section, we describe a potential shortcoming of the method described in Section 2.3.1 for storing a finite-state automaton in a superposition vector. In particular, the method of creating the vectors stored into the superposition vector may introduce dependencies between the vectors that reduces the reliability of the recall. This shortcoming did not affect the experiments reported in this article but could be significant in other situations. To describe the potential shortcoming (below), we will assume that variant ***S1*** is being used which has binarized counters. The interference would also occur in variant ***S2*** but the analysis would be different.

The example given in Section 2.3.1 creates the superposition vector **s** by superimposing together four vectors, each of which encodes a single transition. Since this is an even number of vectors, a random vector will be added to the superposition vector to break ties before it is used for recall. So, for a specific vector stored, there are four other vectors added (which we will call “overlaps”). As will be described in Section 2.4.4.1, if all of the vectors are independent, then the probability of error in one bit when recalling a vector is the probability that none or one out of the four overlaps matches the value of the bit stored. This probability is $\left(\begin{array}{c} 4\\ 0 \end{array}\right)/2^{4}$ = 1/16 (for zero matching) plus $\left(\begin{array}{c} 4\\ 1 \end{array}\right)/2^{4}$ = 4/16 (for 1 matching). The total is 5/16 = 0.3125. However, the actual probability of error when recalling a vector formed using Equation (2) is higher (0.375) because the terms are not independent, as is shown below.

The terms in Equation (2) can be rearranged by collecting common terms as:


(3) $$\begin{array}{l} a=p○l○\rho (l)+t○l○\rho (u)+p○u○\rho (l)+t○u○\rho (u)\\ \;=(p○\rho (l))○(l+u)+(t○\rho (u))○(l+u)\\ \;=(p○\rho (l)+t○\rho (u))○(l+u) \end{array}$$

The first expression in the above equation (**p** ○ *ρ*(**l**) + **t** ○ *ρ*(**u**)) must be either −2, 0, or +2 because it is the sum of two bipolar values {−1, +1}. The possible combinations forming the sum are: 1+1 = 2, 1 − 1 = 0, −1+1 = 0, −1 − 1 = −2. From the possible combinations, it can be seen that the probability of each value is: 2 (1/4), 0 (2/4), and −2 (1/4). These same properties hold for the second expression in the last line of Equation (3), that is: (**l** + **u**). Since the result is the product of these two expression, the product must be either −4, 0 or +4. There are two ways the product can equal four: 2·2 and (−2)·(−2). The chance of each of these is (1/4)·(1/4) = 1/16, so the total probability is 2/16. The probability of the product being −4 is the same (2/16). So the probability that the product is either +4 or −4 is 4/16 and, thus, the probability that the product is zero is 12/16 or 3/4. If the product is zero, then the chance of error is 0.5 because a random vector is added to break ties. So the overall probability of the error is 3/4·1/2 = 3/8 = 0.375.

For the experiments described in this article, this reduction in accuracy did not seem to be significant, probably because the state change combinations that cause the interference occurred infrequently relative to other state transitions that did not cause interference. Such interference could be prevented by using a unique permutation to encode each input instead of a vector.

### 2.4. Predicting the error rate

As described in Sections 2.3.1 and 2.3.2, for both superposition and SDM-based memories the final step of recalling a transition is to compare the recalled noisy next state vector (**r**) to the vectors in the item memory to select the state associated with the vector in the item memory with the closest match. In this section, we present methods we used to predict the error rate of this match, that is the rate for which an incorrect state is selected as the next state. The methods work by calculating the probability of correct recall (*p*_*corr*_) then the error rate is given by 1 − *p*_*corr*_.

#### 2.4.1. High-level equations for probability of correct recall

For all of the memory variations (superposition vector and SDM) the probability of correct recall given a key vector is the probability that the vector obtained from the memory during the recall phase more closely matches the value associated with that key (the matching vector) in the item memory and not one of the other vectors (distractor vectors). Let us assume that **r** is the noisy vector recalled from the memory, **I** is the item memory (an array of vectors), **I**_*m*_ is the matching vector in the item memory, **I**_*d*_ is any distractor vector, *D*(**a**, **b**) is a function returning the distance between two vectors **a** and **b**, and there are *i* vectors in the item memory. The probability of correct recall is then expressed mathematically (adapted from Equation 2.10 in Frady et al., 2018) as:


(4) pcorr=p(D(r,Im)<D(r,Id) ∀d=m)          =∫−∞∞p(D(r,Im)=h)[p(D(r,Id)>h))]i−1dh

#### 2.4.2. Predicting error rate of Hamming distance match memories

If the distance function *D* in Equation (4) is the Hamming distance, then the equation can be re-written as:


(5) $$p_{corr}=\sum_{h=0}^{n}p(H(r,I_{m})=h){\left[p(H(r,I_{d})>h))\right]}^{i-1}$$

where *H*(·, ·) is the Hamming distance between two vectors and *n* is the width of the vectors. In Equation (5), the expression: *p*(*H*(**r**, **I**_*m*_) = *h*) is the “match Hamming distribution” which is the probability mass function for the “match Hamming distance,” that is, the Hamming distance between the vector recalled from the memory (**r**) and the matching vector in item memory (**I**_*m*_). Similarly, expression *p*(*H*(**r**, **I**_*d*_) > *h*) is CCDF_*d*_(*h*) where CCDF_*d*_ is the complementary cumulative distribution function for the “distractor Hamming distribution” (the distribution of Hamming distances between **r** and distractor vectors).

For all of the memories using the Hamming distance match, the distractor Hamming distribution is just the distribution of Hamming distances between two random binary vectors of width *n*_*s*_ (for the superposition vector memory) or *n*_*c*_ (for the SDM variations). This distribution is a binomial distribution *B*(*n, p*) with *n* being the vector width and *p* = 0.5, so the mean is *μ* = *pn* = 0.5*n* and variance is *ρ* = *p*(1 − *p*)*n* = 0.25*n*. So, the distractor Hamming distribution used to calculate *p*(*H*(**r**, **I**_*d*_) > *h*) in Equation (5) is known (given by the binomial distribution just described). What is still needed is the match Hamming distribution, *p*(*H*(**r**, **I**_*m*_) = *h*) for Hamming distances (*h*), in range 0 ≤ *h* ≤ *n*. The determination of this for the different memory variations that use the Hamming distance as the similarity measure are given below in Section 2.4.4.

#### 2.4.3. Predicting error rate for dot product match memories

Memories that use the dot product to determine the similarity between the recalled vector and vectors in item memory use a different representation of vectors and a different operation for the binding. Instead of binary vectors and XOR being used for binding as for the memory variations using the Hamming distance, bipolar vectors are used and binding is implemented by component-wise multiplication. The reason these different methods are used is that the recalled vector in memory variations using the dot product contains integer values (non-binarized counters for the superposition vector or the non-thresholded recalled vector for SDM) and XOR cannot be used as the binding operation with non-binary vectors, but component-wise multiplication with bipolar vectors can.

If the dot product is used as a measure of similarity, Equation (4) can be expressed as:


(6) $$p_{corr}=\int_{-\infty }^{\infty }p(\langle r,I_{m}\rangle =h){\left[p(\langle r,I_{d}\rangle <h)\right]}^{i-1}dh$$

Where 〈·, ·〉 is the dot product between the vector recalled from the memory (**r**) and the vector (either matching or distractor) in item memory. To calculate the error for memories that use the dot product match, it is necessary to calculate the distributions for the dot products for both the case in which the vector in item memory is the correct match to the recalled vector (the match distribution, *p*(〈**r**, **I**_*m*_〉 = *h*)) and also for the case in which the vector in item memory is not the correct match to the recalled vector (the distractor distribution, *p*(〈**r**, **I**_*d*_〉 = *h*)). Equations (5) and (6) cannot be solved analytically but can be calculated numerically if the distributions are known.

#### 2.4.4. Predicting the error rate for each memory variant

Here, we describe how the error rate for each memory variant can be predicted using Equations (5) and (6) and the analysis given above.

##### 2.4.4.1. *S1*

Superposition vector memory ***S1*** uses the Hamming distance between the recalled binary vector and the vectors in item memory to select the closest match. To calculate the probability mass function for the match Hamming distance, the binomial distribution is used twice: first to calculate the probability of error in a single bit in the recalled vector, and second, to calculate the distribution for the match Hamming distance.

If there are *k* vectors added to form the superposition vector (*k* is always odd as described in Section 2.1.1), then the probability that an individual component (bit) in the recalled vector **r** will be in error (that is, not match the corresponding bit of the stored vector being recalled) is the probability that fewer than (*k* − 1)/2 of the other values added to form the superposition match the target value. This probability (which is the expected value of the normalized Hamming distance, symbol *δ*_*s*_) is the CDF of the binomial distribution *B*(*n, p*) with *n* = *k* − 1, *p* = 0.5 evaluated at (*k* − 1)/2 − 1. It is approximated by Kanerva (1997):


(7) $$\begin{array}{l} \delta_{s}=0.5-0.4/\sqrt{k-0.44}. \end{array}$$

The expected normalized Hamming distance (*δ*_*s*_) is the chance that one bit in the recalled vector will not match the corresponding bit in the matching item memory vector. The probabilities of each of the possible Hamming distances to the match vector (that is, the match Hamming distribution in Equation 5) is a binomial distribution, *B*(*n, p*) with *n* = *n*_*s*_, *p* = *δ*_*s*_. The mean of the distribution is *μ* = *pn* = *δ*_*s*_*n*_*s*_ and variance *ρ* = *p*(1 − *p*)*n* = *δ*_*s*_(1 − *δ*_*s*_)*n*_*s*_. Since *n* is large, both the match and the distractor Hamming distributions may be approximated by normal distributions and integration (Equation 4) can be used instead of summation (Equation 5).

##### 2.4.4.2. *S2*

Superposition vector ***S2*** uses non-binarized components in the superposition vector and the similarity between the recalled vector and vectors in item memory is found using the dot product. As described in Section 2.4.3, in order to calculate the recall error rate it is necessary to calculate the distributions for the distance from the recalled vector to both the matching vector in the item memory and a distractor vector. Once obtained, these distributions can be used to calculate the error rate using Equation (6).

To determine these distributions we follow the analysis presented in the Appendix of Gallant and Okaywe (2013). This analysis uses the property that the sum of independent random variables is a random variable with mean equal to the sum of the means and variance equal to the sum of the variances. Since the counter sum consists of the bit for the matching vector {−1, +1} plus the sum of *k* − 1 bits from other vectors, the mean will be the bit value from the matching vector since the mean of the other values is zero. The variance will be *k* − 1 since the variance of the data bit is zero and the variance of the others is 1. The value of the product of the sum with a bit in the matching vector will have mean 1 (since if the bit is 1 or −1, the product will be 1) and the same variance (*k* − 1). The sum forming the dot product will have mean *n*_*s*_ and variance *n*_*s*_(*k* − 1) since it is the sum of *n*_*s*_ random variables, each with mean 1 and variance *k* − 1. Since it is formed by the sum of independent random variables, the central limit theorem applies so the match distribution can be approximated by a normal distribution with mean *n*_*s*_ and variance *n*_*s*_(*k* − 1). The distribution for the distractor distance is determined using the same reasoning, except since the dot product is with a random vector, the mean of the counter sum times a random bit will be zero, and the variance is *k*; so the distribution for the distractor distance is a normal distribution with mean 0 and variance *n*_*s*_*k*.

##### 2.4.4.3. Memories *A1-A4*

To predict the accuracy of the SDM variations ***A1***, ***A2***, and ***A4*** (but not ***A3***) we used computational methods, which calculate the probabilities of different numbers of overlaps onto activated rows in the SDM's contents matrix and from these estimate the match and distractor distributions which can be used in Equations (5) and (6). We did not find a method to predict the accuracy of variant ***A3***. However, we discovered that empirically, for the same number of rows in the SDM's contents matrix, memory variations ***A1*** and ***A3*** had very close to the same error rate. So we used the prediction of error rate for ***A1*** as also the predicted error rate for ***A3***. The details of the methods we used to predict the accuracy of the SDM variations are given in the Supplementary material.

## 3. Results

### 3.1. Memory dimensions for different error rates

The error rates of the different memory variations when storing and recalling a fixed number of vectors is determined by the dimension of the memory, where the dimension of superposition vectors is the width of the vectors (*n*_*s*_) and the dimension of the SDM variations is the number of rows (*m*) in the SDM. To compare the efficiency of the different memory variations for storing data we estimated the dimension required for each variant to store and recall the transitions in a random large finite-state automaton at nine different error rates. Each finite-state automaton was randomly generated and contains 100 states, 10 inputs, and 10 transitions per state. This makes the total number of transitions equal to 1,000 which is the number of vectors that must be stored and recalled from a memory (either superposition vector or SDM). Each automaton has two item memories, one containing vectors corresponding to each state (100 vectors), and one containing a vector for each input symbol (10 vectors). There are a total of 110 vectors in item memory. The error rates that we used for comparing the memory variations were inverse powers of ten, (10^−*r*^) where *r* is an integer in the range 1 to 9 inclusive.

To estimate the dimension of each memory variant that resulted in each of the different error rates when storing the finite-state automaton, we used the methods described in Section 2.4 to predict the error rate of a memory variant of a particular dimension, and we varied the dimension using a binary search to find the dimension that resulted in the closest match to each of the desired error rates. The results of this process are shown in Table 2.

**Table 2:** Estimated memory dimensions to achieve different error rates.

**Error rate**	***S1***	***S2***	***A1***	***A2***	***A3***	***A4***
10^−1^	24002	15221	51;1	50;1	51;1	31;1
10^−2^	40503	25717	86;2	97;1	86;2	57;1
10^−3^	55649	35352	125;2	158;3	125;2	82;1
10^−4^	70239	44633	168;2	208;3	168;2	101;2
10^−5^	84572	53750	196;3	262;3	196;3	129;2
10^−6^	98790	62795	238;3	322;3	238;3	160;2
10^−7^	112965	71812	285;3	368;5	285;3	177;3
10^−8^	127134	80825	311;4	425;5	311;4	205;3
10^−9^	141311	89843	357;4	486;5	357;4	238;3

The error rate is the probability that an incorrect vector is selected as the matching vector in the item memory when a vector is recalled from the memory variant. The dimensions for the superposition vector memories (***S1*** and ***S2***) are the vector widths (*n*_*s*_). The dimensions for the SDM variations (***A1***–***A4***) are the number of rows in the SDM (*m*), then a semicolon and the number of rows activated (*m*_*a*_) during the storage and recall phases.

To test if the estimated dimensions for the different memory variations resulted in the error rate that was predicted for storing the large finite-state automaton, we implemented these memory variations and simulated storage and recall phases, which allowed measuring the error rate empirically.

Two measurements of error rate were made. The first, which we call the “empirical count error rate” is the number of errors (incorrect state selected in the item memory) per recalled vector. This is the most direct measure, but requires a large number of trials in order to create enough errors if the error rate is small. To match the error rate predicted by Equations (5) and (6) (which have a strict inequality) if there were multiple closest matches in item memory to the recalled vector, this was counted as an error even if the correct matching vector was among the closest matches.

The second method, called the “empirical PMF error rate” empirically determines the probability mass function (PMF) for the distance to the matching vector in the item memory and also to the closest distractor. These distributions are then used to calculate what the empirical error rate would be assuming that the distributions do not change between each recall. The accuracy of the PMF error rate can be determined by comparing the PMF error rate to the count error rate in the range for which enough errors occur to allow accurate estimation of the count error rate.

**Figure 3:**
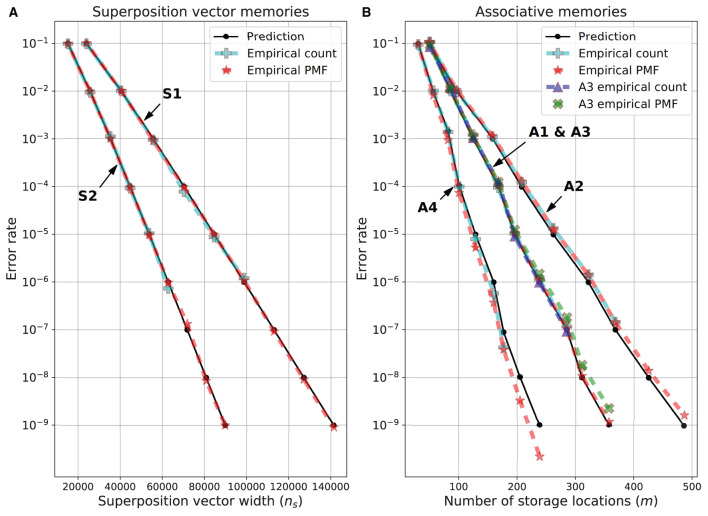
Predicted and empirical error rate vs. dimensions for memory variations. **(A)** Superposition vectors (***S1*** and ***S2***). **(B)** SDM variations ***A1***-***A4***. The dimensions of each memory variation for each error rate are given in Table 1.

The results of these empirical tests are shown in Figure 3. For the superposition vector experiments (Figure 3A), the empirical count error was obtained through an error rate of 10^−6^ and closely matched the predicted error rate. At smaller error rates, it took too long to run the necessary number of simulations to get an accurate count error estimate. However, for these and all error rates the empirical PMF error is in good agreement with the predicted error rate. For the SDM variations (Figure 3B), the empirical count error was obtained through an error rate of 10^−7^. For memory variations ***A1*** and ***A3***, there was a good agreement between the empirical count and the predicted error rates. The ***A1*** empirical PMF error rate closely matched the predicted value for all error rates but the ***A3*** PMF error rate was slightly higher than the predicted values. The ***A2*** empirical PMF and empirical count error rates agreed, but for error rates less than 10^−3^ they were slightly higher than the predicted value, but still close to it. Variant ***A4*** had the biggest difference between the predicted and empirically found error rates—both the empirical count and empirical PMF were lower than the predicted error rate for error rates less than 10^−4^. Despite this, the predicted dimensions for ***A4*** were always less than 10% different from the empirically found dimension for the same error rate, thus, the predicted dimensions are sufficiently accurate for the purpose of comparing the performance of the different memory variations.

### 3.2. Storage required

In this section, we use the estimated dimensions of the different memory variations required to attain particular error rates (cf. Table 2) to compare the storage (number of bits) required for each memory variant to store the large finite-state automaton at each error rate. For each memory variant, the storage required consists of two types of bits: “fixed bits” that contain fixed random values that are compared to vectors being stored or recalled, and “counter bits” that contain counters or the result of binarizing counters. For the superposition vector memories, the fixed bits are the item memories and the counter bits are the superposition vector itself. For the SDM variations, the fixed bits are the address matrix and the item memories, and the counter bits are the SDM's contents matrix.

Depending on how a memory is implemented, the fixed bits may, or may not, be completely present. For example, because vectors in the item memories and SDM's address matrix consist of fixed random bits, it would be possible to implement them in a manner that does not store them explicitly, but instead generates them when needed using a pseudorandom number generator (Kleyko and Osipov, 2017; Schmuck et al., 2019; Eggimann et al., 2021; Kleyko et al., 2022b).

Since (depending on the implementation) the fraction of the fixed bits physically present could range from zero (none physically present) to one (all physically present), to facilitate comparing the memory with different fractions present, we define a variable named “*f*_imp_” (stands for “fraction of the item memory present”) which is in [0, 1] and has the fraction of fixed bits present. We use this variable to compare the storage required for the different memory variations in three cases: if no fixed bits are present (*f*_imp_ = 0), if all fixed bits are present (*f*_imp_ = 1) and for a particular error rate (10^−6^) if the fraction of fixed bits present ranges from none to all (0 ≤ *f*_imp_ ≤ 1).

To calculate the storage for each superposition vector memory the following formula is used:


(8) $$\begin{array}{l} s_{s}=n_{s}\left(f_{\text{imp}}i_{t}+b_{c}\right), \end{array}$$

where *s*_*s*_ is the total storage (in bits) of the superposition vector memory, *i*_*t*_ is the total number of vectors in the item memories (that is 110), *b*_*c*_ is the number of bits per component in the superposition vector (1 for ***S1*** and 8 for ***S2***) and *n*_*s*_ is the width (number of components) of all of the vectors (see Table 2).

The storage of the SDM variations is calculated using:


(9) $$\begin{array}{l} s_{a}=m\;n_{c}\;(b_{c}+f_{\text{imp}})+i_{t}f_{\text{imp}}\;n_{c}, \end{array}$$

where *s*_*a*_ is the total storage (bits) taken up by the SDM; *m* is the number of address locations; *n*_*c*_ is the width of rows in the address and contents matrices as well as vectors in the item memories; and *b*_*c*_ is the number of bits in each counter of the contents matrix. For memory variations using the non-binarized contents matrix (***A1*** and ***A4***) *b*_*c*_ was set to 8 (each counter was one byte) and for variations using the binarized contents matrix (***A2*** and ***A3***) *b*_*c*_ was set to 1 because bipolar values {−1, 1} could be stored using one bit, e.g., {0, 1} with 0 representing −1. For all memory variations *n*_*c*_ was set to 512.

**Figure 4:**
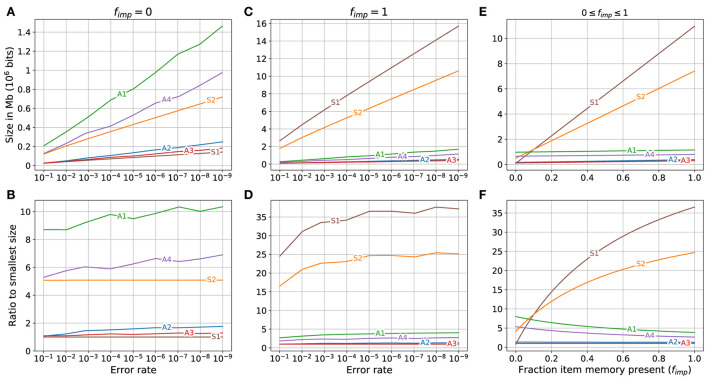
Storage required for different memory variations when different fractions of fixed bits (item memory and SDM's address memory) are present. **(A, C, E)** Top row shows the storage required. **(B, D, F)** Bottom row shows the ratio between the storage required by each variant and that used by the variant occupying minimal storage. Left column: fixed bits (item memory and SDM's address memory) are not present (*f*_imp_ = 0). Middle column: fixed bits are fully present (*f*_imp_ = 1). Right column: Storage required for different memory variations for error rate of 10^−6^ when fraction of fixed bits present ranges from 0 to 1 (0 ≤ *f*_imp_ ≤ 1).

The comparison of storage required for the different memory variations is shown in Figure 4. The case of fixed bits (item memory and SDM's address matrix) not physically present is shown in Figures 4A, B. The three memory variations that use binarized counters (***S1***, ***A3***, and ***A2***) use the least amount of storage, with ***S1*** using the least followed by ***A3*** and ***A2***. The three memory variations that use non-binarized counters (***A1***, ***A4*** and ***S2***) require the most amount of storage, with ***A1*** using the most. There is a clear separation between the three using the least, and the three using the most.

The storage required for the different memory variations if the fixed bits are fully present (*f*_imp_ = 1) is shown in Figures 4C, D. The SDM variations (***A3***, ***A2***, ***A4*** and ***A1***) use the least amount of storage in that order. The superposition vectors (***S1*** and ***S2***) require the most storage with ***S1*** requiring substantially more than ***S2***. Both of the superposition vectors require much more storage than the SDM variations. The storage used by superposition vector memories ***S1*** and ***S2*** are, respectively: 25 to 35, and 15 to 25 times larger than that used by the smallest SDM variant (***A3***), while the storage used by the other SDM variations is less than five times that used by ***A3***. The largest SDM variant (***A1***) uses less than a fifth of the storage used by the smallest superposition vector (***S2***).

For a specific error rate (10^−6^) the storage required for the different memory variations as the fraction of the fixed bits present varied from zero (no fixed bits present) to one (all fixed bits present), i.e., 0 ≤ *f*_imp_ ≤ 1, is shown in Figures 4E, F. On the left side of the graphs, when *f*_imp_ = 0 the superposition vector memory ***S1*** uses less storage than the other memory variations and ***S2*** uses less storage than two of the SDM variations (***A1*** and ***A4***). As the fraction of the fixed bits present increases (toward the right of Figures 4E, F) the increase in the storage required by the SDM variations is minimal compared to the increase in the storage for the superposition vectors causing all of the SDM variations to be much smaller than the superposition vector sizes on the right side of the graphs.

**Figure 5:**
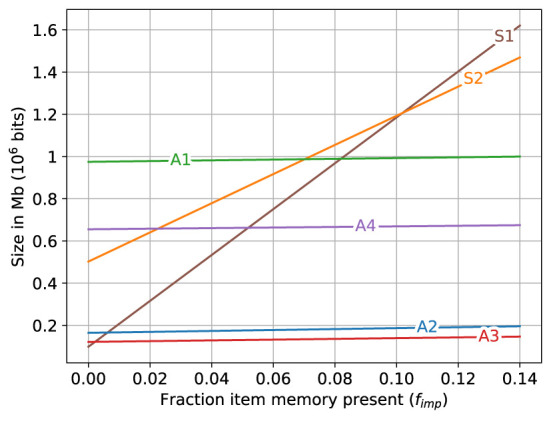
A zoomed view of lower left of Figure 4E. The storage required for different memory variations when fraction of fixed bits (item memory and SDM's address memory) present ranges as 0 ≤ *f*_imp_ ≤ 0.14.

A zoomed in view of the lower left corner of Figure 4E is shown in Figure 5. The size of superposition vector ***S1*** is smaller than all other memory variations only when *f*_imp_ is less than about 0.005 (0.5%). When *f*_imp_ is about 0.01 (1%), the SDM variations ***A2*** and ***A3*** already require less space than ***S1*** and when *f*_imp_ is about 0.08, the largest associative memory variant (***A1***) approximately matches the size of the smallest superposition vector (***S1***), and for *f*_imp_ larger than that, all of the sizes of the SDM variations are smaller than all of the superposition vector sizes.

Note that while the graphs in Figures 4E, 5 are for the error rate 10^−6^, the graphs for all other error rates look very similar to these except for a change in the scale of the y-axis.

### 3.3. Computations required

The previous Section (3.2) compared the storage (number of bits) required to store a fixed amount of data using each memory variant so that it can be recalled at different error rates. In this section, we compare the number of computations required to recall a vector from each memory variant at the different error rates. To do this we first derive equations giving the approximate number of computations (operations on vector components, i.e., addition, XOR, multiplication and permutation) that must be performed for recalling a vector from each memory variant as a function of the memory dimension. We then use the dimensions given in Table 2 as input to the derived equations to estimate the number of operations required for each memory variant to recall a vector from the stored large finite-state automaton at the specific error rate. We also compare the estimated number of operations to the empirically observed computation time required to recall vectors in a software implementations of the memory variations. Lastly, we compare the number of computations required to recall vectors for the different memory variations if operations that are performed on vectors are done in parallel.

To derive the equations for the number of operations, each computation that is performed on a scalar value is assumed to require one operation, except for the computations used to search for the closest match in the state item memory, which is done using either the Hamming distance or the dot product. The computations performed to find the Hamming distance are thresholding a counter and computing the XOR between the thresholded value and the corresponding bit of the vector in item memory. The computations performed for the dot product are to multiply the counter sum by the bit {−1, +1} in the item memory. It was determined empirically that the computation required for the dot product (multiplication) require about 3.4 more time than the computations required for the Hamming distance. To allow a single equation to be given for both cases, we incorporate a variable *q* which equals 1 for the Hamming distance calculation and 3.4 for the dot product calculation.

For the superposition vector memories, the computations performed when recalling the next state vector are (i) release the state and input vectors from the superposition vector (assumed number of operations: 2*n*_*s*_); (ii) permute the result of binding (assumed number of operations: *n*_*s*_); (iii) compute the Hamming distances (***S1***) or dot products (***S2***) between the result vector and each vector in the state item memory (assumed number of operations: (*qn*_*s*_ + *n*_*s*_)*i*_*s*_, where *i*_*s*_ is the number of vectors in the state item memory, i.e., 100)^3^; and (iv) select the closest match to the result vector (assumed *i*_*s*_ operations). Thus, the total number of operations is:


(10) $$\begin{array}{l} c_{s}=2\;n_{s}+n_{s}+\left(q\;n_{s}+n_{s}\right)i_{s}+i_{s}\\ \;=n_{s}\;(3+i_{s}\left(q+1\right))+i_{s} \end{array}$$

where *c*_*s*_ is the number of operations for recalling a vector and finding the closest match in item memory for variant ***S1*** (*q* = 1) and ***S2*** (*q* = 3.4). This equation does not include operations that could be used to generate the item memory if the item memory is created when needed instead of being stored (*f*_imp_ < 1, as described in Section 3.2).

For the SDM variations, the computations performed when recalling a vector are: (i) compute the address vector **a** by binding the current state and input vectors (assumed number of operations: *n*_*c*_); (ii) find the Hamming distance between the address vector and each hard location label in the SDM (assumed number of operations: *mn*_*c*_); (iii) select the *m*_*a*_ locations with the smallest Hamming distance (assumed number of operations: *mm*_*a*_)^4^; (iv) compute the sum of counters in each column of the *m*_*a*_ activated rows (assumed number of operations: *m*_*a*_*n*_*c*_); (v) release the address vector **a** from the sum to form the permuted noisy next state vector (assumed number of operations: *n*_*c*_); (vi) inverse permute the permuted noisy next state vector to form the recalled vector (assumed number of operations: *n*_*c*_); (vii) compute the Hamming distances (for variants ***A1*** and ***A2***) or dot products (variants ***A3*** and ***A4***) between the recalled vector and vectors in the item memory (assumed number of operations: (*qn*_*c*_ + *n*_*c*_)*i*_*s*_); and (viii) select the closest match to the result vector (assumed *i*_*s*_ operations). Thus, the total number of operations is:


(11) $$\begin{array}{l} c_{a}=n_{c}+m\;n_{c}+m\;m_{a}+m_{a\;}n_{c}+n_{c}+n_{c}+\left(q\;n_{c}+n_{c}\right)i_{s}+i_{s}\\ \;=n_{c}\;(3+m+m_{a}+i_{s}\left(q+1\right))+m\;m_{a}+i_{s} \end{array}$$

where *c*_*a*_ is the number of operations for recalling a vector and finding the closest match in item memory for memory variations ***A1*** and ***A2*** (*q* = 1) as well as variations ***A3*** and ***A4*** (*q* = 3.4).

#### 3.3.1. Serial computations required

**Figure 6:**
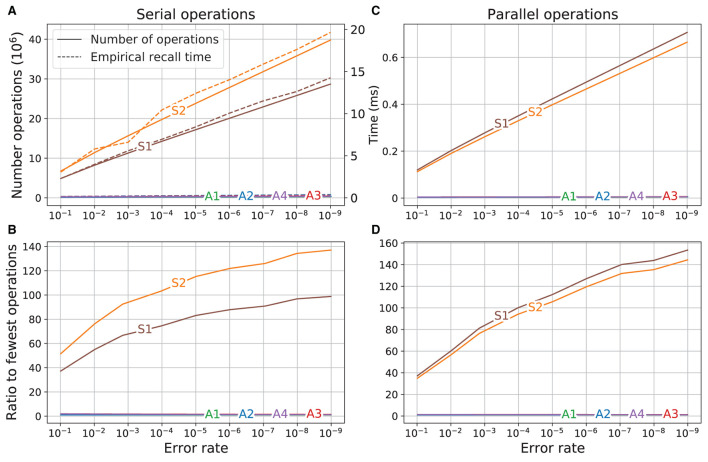
Comparison of operations and run time. Left: operations are serial; Right: operations are parallel. **(A)** Number of operations (solid line, left axis) and empirical time (dashed line, right axis). **(B)** Ratio of number of operations to operations of variant using the fewest operations. **(C)** Number of parallel operations. **(D)** Ratio of number of parallel operations to variant using fewest operations.

The result of using the dimensions of the memory variations for the different error rates (see Table 2) as input to Equations (10) and (11) to calculate the number of operations required to recall a vector for the different memory variations and the corresponding empirically found computation times are shown in Figure 6A. The empirical times were scaled so that the time for variant ***S1*** at error rate 10^−1^ corresponds to the number of operations of ***S1*** at that error rate. There was a good qualitative agreement between the number of operations calculated for each memory variant and the empirical computation time. The time required for both of the superposition vectors (***S1*** and ***S2***) was always much higher and increased much faster as the error rate was reduced than that for every SDM variant, which all required a comparatively constant time for the different error rates.

The ratios between the number of operations for each variant to the number of operations used by the most efficient variant (***A1***) is shown in Figure 6B. On the left side of the graph (error rate 10^−1^), the superposition vectors ***S1*** and ***S2*** required respectively about 37 and 51 times as many operations as did variant ***A1***. As the error rate was reduced (direction to the right of the graph), the difference between the computations required for the superposition vectors and SDM variations increased. At the lowest error rate (10^−9^), the number of computations required by ***S1*** and ***S2*** were, respectively, about 100 and 135 times higher than that of ***A1***.

#### 3.3.2. Parallel computations required

The empirical computation times shown in Figure 6A were obtained from a program running on a computer that was not performing vector operations in parallel. Here we estimate how much faster the computations could be done if the operations that are performed on multiple vectors were done in parallel, so that the time required to perform the operation on a group of vectors was equal to the time required to perform the operation on a single vector.

To do this, we derive alternatives to Equations (10) and (11) (which give the number of operations required to recall a vector from the different memory variations) by replacing each assumed number of operations that is a computation on a group of vectors with the number of operations used for a single vector. For the superposition vector, the specific change is in step (iii): find the distance between the result vector and each vector in item memory. The assumed number of operations ((*q* *n*_*s*_ + *n*_*s*_) *i*_*s*_) is changed to: (*q* *n*_*s*_ + *n*_*s*_) (all matches to item memory are done in parallel). For the SDM variations, the specific changes are in steps: (ii) find the Hamming distance between the address and each hard location label in the SDM; the assumed number of operations (*m* *n*_*c*_) is changed to *n*_*c*_; (iv) compute the sum of counters in each column of the *m*_*a*_ activated rows; the assumed number of operations (*m*_*a*_ *n*_*c*_) is changed to *m*_*a*_; (vii) compute the Hamming distances (***A1*** and ***A2***) or dot products (***A3*** and ***A4***) between the recalled vector and vectors in item memory. The assumed number of operations ((*q* *n*_*c*_ + *n*_*c*_) *i*_*s*_) is changed to (*q* *n*_*c*_ + *n*_*c*_).

With the above changes, the modified equations, which give the number of operations required if operations performed on more than one vector are counted as a single operation, are:


(12) $$\begin{array}{l} c_{s}^{\prime }=2\;n_{s}+n_{s}+q\;n_{s}+n_{s}+i_{s}\\ \;=n_{s}\;(4+q)+i_{s}; \end{array}$$

(13) $$\begin{array}{l} c_{a}^{\prime }=n_{c}+n_{c}+m\;m_{a}+m_{a}+n_{c}+n_{c}+q\;n_{c}+n_{c}+i_{s}\\ \;=n_{c}\left(5+q\right)+m_{a}\left(m+1\right)+i_{s}, \end{array}$$

where $c_{s}^{\prime }$ and $c_{a}^{\prime }$ are, respectively, the number of parallel operations (operations that are in parallel if possible) for recall using the superposition and SDM variations. The plot of the number of operations using these equations is shown in Figure 6C. Having parallel operations reduces the number of operations required (as evident in the change in scale between Figures 6A, C), but the slopes of the curves remain similar, which means that the SDM variations still use far fewer operations than the superposition vector memories.

The ratios of number of operations are shown in Figure 6D. At error rate 10^−1^ the superposition vectors required about 35 times as many operations as did variant ***A1***. This factor increased as the error rate was reduced, so at error rate 10^−9^, the superposition vector memories require about 150 times the number of computations required by variant ***A1***.

## 4. Discussion

### 4.1. General discussion

Computing with vectors is based on three operations: addition, component-wise multiplication (XOR for binary vectors) and permutation of vector components. They allow multiple vectors to be combined “holographically” and superimposed in a single vector of the same width, and to decode such composite vectors.

Such a vector can be used as a memory for values that are accessed with keys (their vectors, that is). The memory is formed by binding the keys to their values with multiplication and adding together the vectors for the key-value pairs. The sum vector is then queried with a vector for a key, and the answer is the value vector for that key, plus noise that can make the memory unreliable.

When using a superposition vector to store key-value pairs in this way, the more pairs that are stored, the greater the chance that individual components of the vector recalled will be different from the corresponding bits in the value. This can be seen easily when analyzing the probability of agreement between the individual components of the superposition vector and its input vectors (see Kleyko et al., 2017 for an example of such analysis).

To overcome the increased probability of mismatch for each bit, the width of the superposition vector and the vectors in the item memory must be increased. This results in more resources (storage space and computations) being required to implement the system.

We investigated how an alternative method for storing and recalling vectors compares to a superposition vector when performing the same task. The alternative method that we used is an associative memory for vectors, specifically variations of the Sparse Distributed Memory (Kanerva, 1988, 1993). Although the idea that the superposition vector shall be used as a working memory while the Sparse Distributed Memory is suitable to implement the long-term memory has been expressed previously, e.g., by Emruli et al. (2015), no studies have been done to quantitatively compare these two alternatives. Also in a vein similar to this study, Steinberg and Sompolinsky (2022) examined how sets of key-value pairs represented with HD computing can be stored in associative memories using a Hopfield network. In Steinberg and Sompolinsky, however, the main focus was on the aspect of using HD computing for flexibly forming fixed-length distributed representations. This aspect can, nevertheless, be interpreted as a way of using the superposition vector to form a working memory.

We compared the storage space and computations required by two variations of a superposition vector memory (***S1***, and ***S2***) and four variations of Sparse Distributed Memory (***A1***-***A4***) in the storage and retrieval of a finite-state automaton containing 1,000 vectors with 110 vectors in item memory. All of the memory variations store data by adding bipolar vectors to vectors of counters. In the superposition vector memories, there is only one vector of counters, while the Sparse Distributed Memory variations have multiple vectors of counters. In half of the memory variations (***S1***, ***A2*** and ***A3***), the counters are binarized (converted to 1-bit) after the data is stored and before recall; in the others, the counters are not binarized. Two of the associative memory variations (***A1*** and ***A2***) threshold the sums of counters before the match to item memory, the other two do not.

In order to compare the memory variations, we first devised methods to predict the error rate of recall of each variant given the number of items stored and the dimensions of the memory (width of the superposition vectors and number of locations in the associative memories) and the item memory size. We used these methods to estimate the dimensions of the memory needed to store and recall the finite-state automaton at different levels of reliability. These dimensions were then used to compare the storage space and computations required of the memory variations at the different error rates. When devising the methods, we found that the dimensions for associative memory variations ***A1*** and ***A3*** were approximately the same for the same error rate. In variant ***A1***, the counters are not binarized but the sums are thresholded; in variant ***A3*** the counters are binarized but the sums are not thresholded.

The storage space required by each memory variant depends on the fraction of item memory (and address locations in the Sparse Distributed Memory) that is explicitly represented and the recall error rate. If none of the item memory is explicitly represented, the three memory variations that use binarized vectors to store data use much less space than the others, with ***S1*** using the least. As the fraction of item memory explicitly represented increases, the space required by the superposition vectors increases much faster than the space required by the Sparse Distributed Memory variations, so that when just 10% of the item memory is explicitly represented, all of the Sparse Distributed Memory variations use less storage space than the two superposition vector variations. When the item memory is fully present, the superposition vectors memories require from 15 to 35 times the space used by the smallest associative memory and all associative memories use less than a fifth of the space than either of the superposition vector memories.

The comparison of computations showed that the superposition vector memories required about 37 and 51 times as many operations to recall a vector as the most efficient associative memory when the error rate of recall was high (0.1). As the error rate is reduced, the superposition vector memories become even less efficient relative to the associative memories. At recall error rate 10^−9^ the superposition vector memories require 100 and 135 times the number of operations of the most efficient associative memory. If the computations were implemented using operations that work on multiple vectors in parallel, the number of operation is reduced but the associative memory variations still use far fewer operations than the superposition vector memories.

The reason that the Sparse Distributed Memory performs better than a superposition vector for the recall of key-value pairs is due to the difference in how the vector that is compared to the vectors in the item memory (to find the matching value) is generated. For the superposition vector, this vector is generated by multiplying the superposition vector with the key. This generates a vector which is similar to the value, but the probability of a component in this vector matching the corresponding bit in the value is reduced as more key-value pairs are superimposed, and there is no way to improve the probability. With the Sparse Distributed Memory, the vector that is compared to the item memory is generated by summing the activated hard locations in the Sparse Distributed Memory's contents matrix. As with a superposition vector, the probability of a component in this vector matching the corresponding bit in the value is reduced as more key-value pairs are stored in the Sparse Distributed Memory. However, unlike the superposition vector, the probability that each component will match the corresponding bit in the value can be increased by increasing Sparse Distributed Memory capacity (that is, increasing the number of rows in the Sparse Distributed Memory's contents matrix). This allows the width of vectors in the Sparse Distributed Memory to be shorter than the superposition vector while still correctly finding the matching value vector in the item memory. The shorter vectors used with the Sparse Distributed Memory reduces the space required to store the item memory and reduces the number of operations needed for comparison during recall which improves the performance of the Sparse Distributed Memory as compared to a superposition vector.

The performance improvements we describe do not mean that it is always advantageous to use a Sparse Distributed Memory instead of a superposition vector. There are some situations where certain manipulations can be performed using a superposition vector that cannot easily be done with a Sparse Distributed Memory. The most prominent example of such a manipulation is the formation of hierarchical representations (that are also called sketches in some context) when the representation of some compositional data structure (e.g., a set of key-value pairs) is used to form a representation of a larger structure that subsumes the compositional data structure as its part at some level of hierarchy (Plate, 1994a; Rachkovskij et al., 2013; Ghazi et al., 2019). It is straightforward to use the superposition vector to form such hierarchical representations while in the case of a Sparse Distributed Memory one first needs to design a procedure that will convert the relevant part of the memory into a vector representation that can be further manipulated with HD computing.

There were two sets of item memories used to encode the finite-state automaton: one for states and the other for inputs. The vectors recalled from the memories were compared to the state item memory contents, but not the input item memory. If there were multiple item memories, and the vectors recalled had to be compared selectively to vectors in a particular item memory, there would need to be some mechanism to identify which item memory was appropriate to use and circuitry to route recalled vectors to the proper item memory for comparison. The identification of the item memory might be done using codes (bit patterns) that could be included in the vectors to identify them as being of a particular type of item memory. We did not include such methods in the analysis presented in this article.

### 4.2. Connection to psychology and neuroscience

Cognitive psychology likens brains to computers: to physical devices that manipulate information (e.g., Miller, 1956). The encoding of information by brains and the organization of memory have been described in computer-like terms since the 1960s and '70s (e.g., Anderson and Bower, 1967; Bower, 1967; Neisser, 1967; Atkinson and Shiffrin, 1968). For example, information is encoded into chunks in working memory and the chunks are used both as cues to long-term memory and as data to be stored. The data can then be retrieved if the chunks used as the cue are the same as when the data were stored. This has been called “encoding specificity” and has been demonstrated in psychological experiments (Tulving and Thomson, 1973). In computer terms it simply means that stored data can be recalled by knowing the address.

While human long-term memory seems boundless, human short-term memory—referred to here as the working memory—is limited to a mere seven or so items (Miller, 1956). This suggests a two-tier memory organization similar to that of a computer. The computer's working memory comprises an Arithmetic–Logic Unit (ALU) and a handful of registers that are wired for arithmetic and Boolean operations. The computer's long-term memory is an array of registers addressed by their position in the array—it is the RAM (Random Access Memory) that today's computers have billions of bytes of. The contents of the ALU—the working memory—turn over completely millions of times a second whereas the contents of the RAM change hardly at all in comparison. This raises the question, are there brain structures and processes suggestive of computer-like memory and processing? The answer for the long-term memory is “yes” and for the working memory “possibly.”

A computer RAM is a simple and highly regular circuit that has over half the computer's transistors. Among the brain's circuits, the cortex of the cerebellum has over half the brain's neurons (and 5 trillion modifiable synapses). It was interpreted as an associative memory by Marr (1969). Furthermore, its three-dimensional structure corresponds to how an engineer would build a RAM-like memory from neuron-like components (Albus, 1971; Kanerva, 1993): mossy fibers (200 million) as address lines, granule cells (50 billion) as memory locations, climbing fibers (15 million) as data-input lines, and Purkinje cells (15 million) providing the output—the numbers in parentheses are estimates for the human cerebellum. A recent study shows that the small number inputs to a cerebellar granule cell, and the large number of inputs to a Purkinje cell, are optimal for an associative memory (Litwin-Kumar et al., 2017). Brains may have other long-term memory structures as well, including the fly olfactory system (Dasgupta et al., 2017), but none that is as large and equally well-understood.

The issue with working memory is more complicated because no brain structure is an obvious candidate. However, we can conjecture its nature by analogy to the computer's working memory (the ALU). The ALU takes a pair of numbers as input and outputs their sum or product or some other arithmetic function, and it operates similarly with logical bit strings. The inputs and the outputs are stored in the RAM. Complex relations and structures can thus be expressed in the long-term memory by combining their constituent parts, piece by piece, in the working memory.

This manner of representing relations and structure is possible also when we compute with high-dimensional vectors (Plate, 1994a), and presumably possible for brains. Three simple operations on vectors are sufficient, besides being readily realizable in neurons. The working memory needs to combine a few vectors at a time and store the result in the long-term memory, and to decode vectors stored therein. The superposition vector provides such a mechanism and its capacity is limited by its width, as discussed above.

The item memory, however, is an engineering artifact. It is long-term, and a (neural) associative memory can fulfill the functions of both the item memory and the more general long-term memory. The engineering of a high-capacity associative memory, however, is a major challenge. We have modeled it here with the Sparse Distributed Memory, but its circuit for selecting memory locations is inefficient and quite different from the cerebellum's. This is clearly an area where a more complete understanding and modeling of the cerebellum can lead to a more efficient engineering design.

Finally, it is worth noting that the finite-state automata that were stored in our experiments could be seen as a model for biological reflexive response to sensory stimuli and also for robotics (Osipov et al., 2017; Neubert et al., 2019).

## Data availability statement

The datasets presented in this study can be found in online repositories. The names of the repository/repositories and accession number(s) can be found below: https://github.com/jeffteeters/hdfsa.

## Author contributions

JLT, DK, PK, and BAO came up with the idea for the study. JLT wrote and ran the programs that generated the figures and did most of the mathematical analysis. DK contributed to the mathematical analysis and also wrote some of the programs used to obtain the results. JLT, DK, and PK wrote the manuscript. BAO supervised the project. All authors contributed to the article and approved the submitted version.

## Footnotes

^1^In fact, a superposition vector could be considered as the special case of SDM with only one storage location that is always activated.

^2^This formula might not give the optimal value of *m*_*a*_ for other variations. If so, with a more optimal value, the SDM may perform even better than what was reported in the experiments described in Section 3.

^3^The second *n*_*s*_ in the expression is present because the result of the *n*_*s*_ component-wise operations—either XOR or multiplication—must be added using approximately *n*_*s*_ sums to calculate the similarity.

^4^If *m*_*a*_>1, the actual number of operations will be less than this depending on the algorithm used. For example, if the *m*_*a*_ smallest values found so far were maintained using a heap, the number of operations would be about *mlog*(*m*_*a*_), since the time to insert a new value into a heap of size *m*_*a*_ is order log(*m*_*a*_).
