# Supplementary material for: On separating long- and short-term memories in hyperdimensional computing
Source: Front Neurosci. 2023 Jan 9;16:867568. doi: 10.3389/fnins.2022.867568 (PMC9869149; doi:10.3389/fnins.2022.867568)
Supplement: Supplementary file 1 [file Data_Sheet_1.pdf]

Supplementary Material

1 METHODS OF PREDICTING ERROR RATE OF SDM ASSOCIATIVE MEMORIES

1.1 Overview

This document (which is referenced in Section 2.4.4.3 in the main text) describes how the error rate for the associative memories (***A1-4***) were predicted. The properties of the associative memories are described in the main text in Section 2.2 and summarized in Table 1 in the main text.

1.1.1 Program code

The descriptions given here are meant to be complete, in that they should allow someone to implement the algorithms just by reading this document. However, in addition, at the top of the description of each method the name of the Python script that implements the method is provided. The scripts are available on GitHub at: `https://github.com/jeffteeters/hdfsa`. The scripts can be used to supplement the descriptions.

1.1.2 Error rate

In the main text, the procedure used for storing, then recalling data from an SDM is described in Section 2.3.2 and the error rate of the recall is described in Section 2.4. To be sure that it is clear what the computations are doing, here we describe in other words what is meant by the "error rate." In the empirical experiments that were done for each memory, key–value pairs were stored in the memory, where the key was the current state of a finite state automaton bound to the input, and the value was the key bound to the next state then permuted. The data is stored in the SDM by using each key as an address vector and the associated value as the data vector. The address vector is used to select m_a of the m rows in the SDM contents matrix and the data vector is stored in the selected rows by incrementing and decrementing the counters. The width of all the vectors is n_c. After all of the key–value pairs are stored, for memory variants ***A2*** and ***A3*** the SDM contents matrix is binarized (converted to $+1$s and -1s) before data is recalled. The data is recalled by iterating through the keys, and each key is used as an address vector presented to the SDM which selects the same m_a rows used to store the data vector and the column-wise sums of counters in the selected rows are computed. The column-wise sums is the recalled data vector ($\mathbf{d}$) for variants ***A3*** and ***A4***, and the column-wise sums are thresholded to form the recalled data vector for variants ***A1*** and ***A2***. The recalled data vector is bound to the key then inverse-permuted to obtain a noisy vector $\mathbf{r}$ which should be more similar to the next-state vector associated with the key than to other state vectors. A measure of similarity (Hamming distance for ***A1*** and ***A2***; dot product for ***A3*** and ***A4***) is calculated between this vector and all of the vectors in the state item memory. If there is only one closest matching vector and if that vector corresponds to the correct next state then the recall was successful. If this is not the case (if there is another vector that is equally close or closer—according to the measure of similarity) that is classified as an error. The error rate is the number of such errors that occur divided by the total number of vectors recalled.

1.2 Variant *A1*

The program code for this is in file `sdm_ae.py`.

The algorithm to calculate the error rate for variation ***A1*** has three steps. Step 1 is to calculate the probability of the most likely configurations of overlaps onto the target rows. Once that is obtained, then,

in Step 2, the single-bit error rate for each of these configuration is computed. These error rates are used in Step 3 to compute the match Hamming distribution which is used in Equation (5) in the main text to calculate the error rate. The details of these steps are given below.

1.2.1 Step 1 - Calculate overlap configuration probabilities

1.2.1.1 Background

If the data vector being recalled is called the "target," the probability that a thresholded sum will be in error (not match the corresponding bit in the data vector) is determined by which rows the other data vectors stored in the memory are stored in. For each other vector stored in the memory, the selected rows used to store that vector may overlap the rows used to store the target (the "target rows") from zero (no overlap) to m_a times (complete overlap). The number of target rows overlapped may be regarded as the "weight" of the overlap since that number multiplied by $+1$ or -1 is added to the counter sum. If there are k vectors stored in the memory (one of which is the target), each possible configuration of overlaps onto the target rows by the remaining $k-1$ vectors may be represented by a tuple q_v^g of integers of length m_a+1, $(q_{v0}^g, q_{v1}^g, q_{v2}^g, ...q_{vm_a}^g)$. The superscript g is an integer that indicates how many vectors other than the target are stored and the subscript v is an integer that distinguishes one configuration from another. The first element in each tuple (q_{v0}^g) is the number of other items that do not overlap the target rows (zero weight overlaps); q_{v1}^g is the number that overlap by one row (weight one), and so on, up to the last element ($q_{vm_a}^g$) which has the number of vectors that have complete overlap (m_a rows overlap, weight m_a). For example, if 999 vectors are stored other than the target ($g = 999$), and $m_a = 3$, one overlap configuration could be $(952, 27, 14, 6)$ which indicates that of the 999 vectors stored, 952 overlapped zero target rows, 27 one row, 14 two rows and 6 three rows. The sum of the overlaps of each weight $(952 + 27 + 14 + 6)$ must equal 999 since every vector stored must overlap either 0, 1, 2 or 3 target rows.

From the length of the tuples $(m_a + 1)$ it is seen that the number of possible configurations is the number of ways of distributing $k-1$ indistinguishable items (other vectors) among m_a+1 distinguishable bins (weights). The expression for this can be found using the "stars and bars" counting method, and is $\binom{k-1+m_a}{m_a}$. If $k = 1{,}000$, then the number of configurations for $m_a = 1, 2, 3, 4$ are respectively about: one thousand, 0.5 million, 167 million, 42 billion. The large numbers if $m_a > 2$ may make it impractical to calculate the probability of all of the possible configurations. To reduce the number of configurations considered, the algorithm (given below) can periodically prune from consideration configurations that are least probable. As will be described in Section 1.2.1.4 the pruning reduces the number of configuration considered for $m_a = 4$ from 42 billion to under 700 while allowing an accurate estimate of the error rate.

Associated with each overlap configuration (tuple q_v^g) is the probability (denoted as p_v^g) of that configuration occurring. For example, one possible configuration if $k = 1{,}000$ and $m_a = 3$ is the tuple $(0, 0, 0, 999)$ which corresponds to all other vectors completely overlapping the target rows. The probability of this can be calculated as follows: The probability of one other vector completely overlapping the target row is the probability that the first row selected when storing that vector is among the target rows (this probability is $3/m$) times the probability that the second row overlaps the remaining two target rows (this probability is $2/(m-1)$) times the probability that the third overlaps the remaining target row (this probability is $1/(m-2)$). This product, $((3/m)\,(2/(m-1))\,(1/(m-2)))$, is the probability of *one* other vector overlapping all the target rows. The probability of *all* other $k-1$ vectors completely overlapping the target rows is that product raised to the power $k-1$.

1.2.1.2 The algorithm

While the probability for some special cases (like the example given above) can be calculated directly, calculating the probability of other configurations, such as the configuration mentioned previously $(952, 27, 14, 6)$, is not as straightforward. The algorithm we used to calculate the overlap configuration probabilities works by first (Part 1) calculating the overlap configurations and probabilities due to *one* other vector, then from that, (Part 2) successively calculating the configurations and probabilities for $2, 3, 4, ...k-1$ other vectors. The algorithm (which has two parts) is given below.

Part 1: Calculate q^1 and p^1—that is, the overlap configurations (q^g) and probabilities (p^g) for *one* other vector being stored in the SDM. (Since one other vector is stored, superscripts g in q^g and p^g are one.) The configurations (q^1_v, with v ranging from 0 to m_a) are set equal to tuples of length $m_a + 1$, with the index of each component i ranging from 0 to m_a and $q^1_{vi} = 1$ if $v = i$ otherwise zero. The probabilities (p^1_v, with v ranging from 0 to m_a) are set equal to the probability of selecting v of the m_a target rows from the total of m rows when m_a rows are selected. This is given by the hypergeometric distribution with $p^1_v = \binom{m_a}{v}\binom{m-m_a}{m_a-v}/\binom{m}{m_a}$.

Part 2: Given the configurations (q^g) and probabilities (p^g) for a particular number (g) of other vectors, calculate the configuration and probabilities for that number of other vectors plus one (q^{g+1} and p^{g+1}). This is done by calculating what happens to the configurations and probabilities by storing an additional vector in the SDM that already has g vectors stored (besides the target). These computations are done by computing the outer component-wise sum of q^1 and q^g and the outer product of p^1 and p^g, then converting the 2-D arrays (result of outer sum and product) to 1-D by concatenating the rows. Then any configurations that are the same are combined by replacing them with just one instance of the configuration and setting the probability to the sum of the probabilities of all instances of the configuration. Removal of least probable configurations ("pruning") is done by finding the largest probability (after combining matching configurations), then removing any configurations that have a probability less than one over a threshold of that probability, where the threshold is a large positive number, e.g. 10^7. This part (Part 2) is repeated $k-2$ times, so the end result is the configurations (q^{k-1}) and probabilities (p^{k-1}) for $k-1$ other vectors stored in the SDM.

1.2.1.3 An example

Here the algorithm is illustrated using an example where each key activates two rows of the SDM ($m_a = 2$).

In Part 1, the overlap configurations for one overlap (q^1) are set to: $q^1_0 = (1, 0, 0)$, $q^1_1 = (0, 1, 0)$ and $q^1_2 = (0, 0, 1)$. These respectively are the configurations with zero, one, and two rows overlapping the target rows. The probabilities corresponding to each of these configurations is the chance of selecting without replacement $v = 0, 1$ and 2 target rows from a total of m rows in a sample of size m_a ($m_a = 2$). These probabilities, (p^1_v, i.e., p^1_0, p^1_1, p^1_2) are given by the Hypergeometric distribution $p^1_v = \binom{m_a}{v}\binom{m-m_a}{m_a-v}/\binom{m}{m_a}$.

In Part 2, first the outer component-wise sum of q^1 and q^g and the outer product of p^1 and p^g are calculated. For both of these, $g = 1$ for the first added vector. The outer component-wise sum of q^1 and q^1 is shown in Table S1 and the outer product of p^1 and p^1 ($g = 1$) is shown in Table S2.

Converting the produced 2-D arrays in Tables S1 and S2 to 1-D by concatenating the rows, then aligning the resulting 1-D arrays gives the aligned arrays shown in Table S3. In that figure, there are 9 columns which arise from the 9 elements in the 3×3 matrices. At the top, the index (v) of each configuration is given; v ranges from 0 to 8.

Table S1: Outer sum of configurations (q^1 and q^1).

	$(1,0,0)$	$(0,1,0)$	$(0,0,1)$
$(1,0,0)$	$(2,0,0)$	$(1,1,0)$	$(1,0,1)$
$(0,1,0)$	$(1,1,0)$	$(0,2,0)$	$(0,1,1)$
$(0,0,1)$	$(1,0,1)$	$(0,1,1)$	$(0,0,2)$

Table S2: Outer product of p^1 and p^1.

	p^1_0	p^1_1	p^1_2
p^1_0	$p^1_0\,p^1_0$	$p^1_0\,p^1_1$	$p^1_0\,p^1_2$
p^1_1	$p^1_1\,p^1_0$	$p^1_1\,p^1_1$	$p^1_1\,p^1_2$
p^1_2	$p^1_2\,p^1_0$	$p^1_2\,p^1_1$	$p^1_2\,p^1_2$

Table S3: Outer sums and products aligned.

Index (v):	0	1	2	3	4	5	6	7	8
q^2_v:	$(2,0,0)$	$(1,1,0)$	$(1,0,1)$	$(1,1,0)$	$(0,2,0)$	$(0,1,1)$	$(1,0,1)$	$(0,1,1)$	$(0,0,2)$
p^2_v:	$p^1_0\,p^1_0$	$p^1_0\,p^1_1$	$p^1_0\,p^1_2$	$p^1_1\,p^1_0$	$p^1_1\,p^1_1$	$p^1_1\,p^1_2$	$p^1_2\,p^1_0$	$p^1_2\,p^1_1$	$p^1_2\,p^1_2$

In Table S3, the configurations at $v = 1$ and 3 are the same $(1, 1, 0)$ and the configurations at $v = 5$ and 7 are the same. Combining these multiple configurations that match results in the arrays shown in Table S4.

Table S4: Outer sums and products aligned after combining matching configurations.

Index (v):	0	1	2	3	4	5	6
q^2_v:	$(2,0,0)$	$(1,1,0)$	$(1,0,1)$	$(0,2,0)$	$(0,1,1)$	$(1,0,1)$	$(0,0,2)$
p^2_v:	$p^1_0\,p^1_0$	$p^1_0\,p^1_1 + p^1_1\,p^1_0$	$p^1_0\,p^1_2$	$p^1_1\,p^1_1$	$p^1_1\,p^1_2 + p^1_2\,p^1_1$	$p^1_2\,p^1_0$	$p^1_2\,p^1_2$

Combining the matching configurations reduces the number of components in the q^2 and p^2 arrays from 9 (in Table S3) to 7 (in Table S4) and the probabilities corresponding to the duplicate configurations in Table S3 are aggregated in Table S4.

The end result of one cycle (outer products and outer sum, convert to 1-D, and combine matching configurations) are the configurations and probabilities for 2 vectors stored other than the target (q^2 and p^2). The probabilities for three vectors stored other than the target is formed by the outer component-wise sum (q^1 and q^2) and the outer products of p^1 and p^2. These are, respectively, shown in Tables S5 and S6.

Table S5: Outer component-wise sum of configurations q^1 and q^2.

	$(2,0,0)$	$(1,1,0)$	$(1,0,1)$	$(0,2,0)$	$(0,1,1)$	$(1,0,1)$	$(0,0,2)$
$(1,0,0)$	$(3,0,0)$	$(2,1,0)$	$(2,0,1)$	$(1,2,0)$	$(1,1,1)$	$(2,0,1)$	$(1,0,2)$
$(0,1,0)$	$(2,1,0)$	$(1,2,0)$	$(1,1,1)$	$(0,3,0)$	$(0,2,1)$	$(1,1,1)$	$(0,1,2)$
$(0,0,1)$	$(2,0,1)$	$(1,1,1)$	$(1,0,2)$	$(0,2,1)$	$(0,1,2)$	$(1,0,2)$	$(0,0,3)$

The 3×7 two-dimensional arrays generated by the outer sums and products are converted to 1-D arrays with 21 elements, then like configurations are combined as was done previously. The result are the configurations q^3 and probabilities p^3 for three other vectors stored besides the target. If removing the least probable configurations is done, the largest probability is divided by a specified threshold (e.g., 10^7) and any configurations less than that are removed.

Table S6: Outer product of probabilities p^1 and p^2.

	p_0^2	p_1^2	p_2^2	p_3^2	p_4^2	p_5^2	p_6^2
p_0^1	$p_0^1 p_0^2$	$p_0^1 p_1^2$	$p_0^1 p_2^2$	$p_0^1 p_3^2$	$p_0^1 p_4^2$	$p_0^1 p_5^2$	$p_0^1 p_6^2$
p_1^1	$p_1^1 p_0^2$	$p_1^1 p_1^2$	$p_1^1 p_2^2$	$p_1^1 p_3^2$	$p_1^1 p_4^2$	$p_1^1 p_5^2$	$p_1^1 p_6^2$
p_2^1	$p_2^1 p_0^2$	$p_2^1 p_1^2$	$p_2^1 p_2^2$	$p_2^1 p_3^2$	$p_2^1 p_4^2$	$p_2^1 p_5^2$	$p_2^1 p_6^2$

1.2.1.4 The implementation

To allow for an efficient implementation of the outer component-wise sum of configurations, each configuration (a tuple of length $m_a + 1$) is packed into a single 64-bit unsigned integer of the form: "66,555,444,333,222,111,000" where each block of three digits represents the digits used to store the overlaps of that weight, e.g. the "222" digits store the number of overlaps of weight 2. For example, if $m_a = 3$ and the configuration was the example given in Section 1.2.1.1 $(952, 27, 14, 6)$ the packed integer would be $6{,}014{,}027{,}952$. Storing each tuple as an integer in this way allows a component-wise sum to be performed by just adding two integers, instead of $m_a + 1$ pairs of integers.

This packed representation only works if, in a configuration, there are less than $1{,}000$ overlaps for each weight of 5 or less and the number of overlaps of weight 6 is less than 19.[1] For the results we describe in Section 3.1 of the main text (with $k = 1{,}000$ and with m set to the number of rows which results in a desired error rate—given in Table 2 in the main text—and m_a determined by Equation (1) in the main text) these conditions were always met.

Two thresholds were used to prune the number of configurations, 10^7 and 10^{10}. When $m_a = 4$, the threshold of 10^7 reduced the number of configurations from about 42 billion (if no pruning is done) to less than 700 and the threshold of 10^{10} reduced the number of configurations to less than $1{,}700$. The two thresholds gave similar results when used to estimate the number of rows (m) in the SDM that result in the desired error rates when recalling the stored finite-state automata. The number of rows shown in Table 2 in the main text for variant ***A1*** was generated using the threshold of 10^7. Using the larger threshold (10^{10}) did not change the estimated number of rows, except for the three lowest error rates for which the estimated number of rows was increased by one (e.g., the number of rows for the error rates of 10^{-7}, increased from 285 to 286—a change of less than 0.35%). The pruning did not appear to significantly reduce the accuracy of the calculated match Hamming distributions because there was a good correspondence between the predicted accuracy and the empirical error rates as shown in Figure 3B in the main text.

In the implementation, configurations and probabilities are stored in Python variables as follows: Configurations for one other vector stored (q^1) are stored in a NumPy 1-D integer array named `key_increments` with each tuple (configuration) stored in an unsigned 64 bit integer using the packed representation described above. Configurations for multiple other vectors stored, that is q^g with $g \geq 1$, are stored in variable `cop_key`, which contains 1-D NumPy integer arrays giving the configurations using the packed representations. The probabilities of configurations for one other vector stored (p^1) are stored in a NumPy 1-D float array with variable named `ovl_pmf`. The probabilities of configurations for multiple other vector stored (p^g with $g \geq 1$) are stored in variable `cop_prb`, which contains 1-D NumPy float arrays, each of which has the probabilities for the corresponding configurations in `cop_key`. The

[1] The weight 6 limit is because the maximum value of an unsigned 64-bit integer is 18,446,744,073,709,551,615; the two most significant digits (18) are the maximum value for weight 6 overlaps.

`cop_key` and `cop_prb` arrays go hand-in-hand, because for each overlap configuration in `cop_key` the corresponding element of `cop_prb` has the probability of that configuration.

1.2.2 Step 2 - Calculate single-bit error rate for each overlap configuration

The processing in Step 1 determines the overlap configurations (q^g) and probabilities (p^g) of each configuration due to $k-1$ vectors being stored in the SDM in addition to the target. Each configuration is a tuple of length m_a+1 which gives the number of overlaps of weight 0 through m_a. An example (given previously in Section 1.2.1.1) is the configuration $(952, 27, 14, 6)$ which indicates that of the 999 vectors stored, 952 overlapped zero target rows, 27 one row, 14 two rows and 6 three rows.

In this step, the probability of a single-bit error during recall for each of the configurations is calculated. With the example just given, this would be the probability of a single-bit error if 6 vectors overlapped three target rows, 14 two rows and 27 one row). Each overlap has a 0.5 chance of matching the target bit, so the overlaps add “noise” to the “signal” that is due to the target bit.

The probability of a single-bit error for a configuration is the probability that the thresholded counter sum formed given the configuration will not match the target bit value, that is, the sum will be ≤ 0 when the target bit is one, or the sum will be > 0 when the target bit is zero. If there are no overlaps onto the target rows then the probability of error is zero and no further calculations are needed. Otherwise, the calculation proceeds as follows.

First, the possible sums due to the number of overlaps for each weight greater than zero and the probability of each sum is computed. To do this, two arrays are created for each weight greater than zero in the overlap configuration. The first array (`cw[`w`-1]`) contains all the possible sums due to weight w, with $w>0$. It is set to `cw[`w`-1][`j`]`$= j\,w-(c-j)\,w$ where w is the weight, c is the number of overlaps of that weight, and the index j ranges from 0 up to and including c. The second array (`cp[w-1]`) contains the probabilities of the corresponding sums in the `cw[`w`-1]` array. It is set by `cp[`w`-1][j]`$=\mathrm{B.pmf}(j, c, 0.5)$ which is the probability mass function (PMF) of the binomial distribution for probability of j successes in c trials where the probability of success in each trial is 0.5.

After the possible sums and probability of each sum due to the overlaps for each weight are calculated (as described in the previous paragraph) every possible combination of the sums for the different weights must be added together (and the associated probabilities multiplied) in order to compute the possible overall sums (due to all weights) and their probabilities. The `cw` and `cp` arrays are each an “array of arrays,” because each pair (`cw[`w`-1]` and `cp[`w`-1]`) are 1-D arrays containing respectively all the possible sums and the corresponding probabilities due to the contribution of overlaps of weight w. For example, if $m_a=3$ and the configuration is the example given previously $(952, 27, 14, 6)$ there will be three arrays stored in each: `cw[0]` will be the possible sums due to 1 row overlaps, which is (-27, -25, -23, ... +27); `cw[1]` will be the possible sums due to 2 row overlaps (-28, -24, -20, ... +28); and `cw[3]` will contain the possible sums due to three row overlaps (-18, -12, -6, ..., 18). The corresponding arrays in `cp` (i.e., `cp[0]`, `cp[1]`, `cp[2]`) contain the probabilities of each of the sums. The sums and probabilities in these multiple arrays are combined to form two 1-D arrays that have the possible sums and probabilities due to all the overlaps (for all weights). This is done by iteratively computing the outer sum of the `cw` arrays and the outer product of the `cp` arrays then converting them to 1-D and combining duplicate sums in a manner similar to how the configuration (q^g) and probability (p^g) arrays were processed in the previous step (Section 1.2.1.2 Part 2, and the Example in Section 1.2.1.3). The result of this computation is two 1-D arrays, “wa” which contains all the possible sums for the overlap configuration, and “pa” which contains the corresponding probabilities.

(In the program code the wa and pa arrays are, respectively, stored in variables named `chunk_weights` and `chunk_probabilities`.)

The sums in the wa array are sums due to the overlaps, but do not include the contribution from the target vector. The sums for the overlaps plus the contribution from the target vector (that is the final sums that are thresholded) are found by adding m_a to each element of wa if the target bit is 1, and by subtracting m_a from each element of wa if the target bit is zero. That is, $\mathrm{s}_1 = \mathrm{wa} + m_a$ and $\mathrm{s}_0 = \mathrm{wa} - m_a$ where s_1 and s_0 are, respectively, the possible sums if the target is 1 or 0. The probability of an error when the target bit is 1 (pe_1) is found by summing the probabilities in pa corresponding to sums in s_1 that are ≤ 0. That is: $\mathrm{pe}_1 = \sum \mathrm{pa}[j] \ \forall j \mid (\mathrm{s}_1[j] \leq 0)$. Similarly, the probability of an error when the target bit is 0 (pe_0) is found by summing the probabilities in pa corresponding to sums in s_0 that are > 0. That is: $\mathrm{pe}_0 = \sum \mathrm{pa}[j] \ \forall j \mid (\mathrm{s}_0[j] > 0)$. The final probability of error for the configuration is then calculated as: $\delta_c = (\mathrm{pe}_1 + \mathrm{pe}_0)/(2 \sum \mathrm{pa})$. The division by 2 is done to compute the average and the division by $\sum \mathrm{pa}$ (the sum of the probabilities) is done to be sure that the probability is normalized to the range 0 to 1. (Dividing by the sum of the probabilities may not be necessary because the sum of the probabilities should be close to one anyway.) In the program code pe_1 and pe_0 are, respectively, stored in variables `perror_sum` and `nerror_sum` and the δ_c for each configuration is stored in variable `cop_err`.

1.2.3 Step 3. Calculate match Hamming distribution

The match Hamming distribution is calculated using: $p(h) = \sum_v p_v^g \, \mathrm{B.pmf}(h, n_c, \delta_{cv}) / \sum_v p_v^g$, where h is a specific Hamming distance (h ranges from 0 to n_c inclusive) and both summations are over all configurations (all v) and $\mathrm{B.pmf}(h, n_c, \delta_{cv})$ is the probability mass function of the binomial distribution for probability of h successes in n_c trials where the probability of success in each trial is δ_{cv} which is the single-bit error rate for configuration v. The division by the second summation is done to normalize the calculated probabilities so the sum will be 1 (making it a probability distribution). This is needed in case some of the unlikely configurations were removed from consideration (pruned) in Step 1 because that will cause the sum of the probabilities in p^g to be less than one since the probabilities of configurations that were pruned are not included in the sum. This probability mass function is then used in Equation (5) in the main text for $p(H(\mathbf{r}, \mathbf{I}_m) = h)$ (the match Hamming distribution). In the program code, $p(h)$ is stored in variable `hist`.

1.3 Variant *A2*

Associative memory ***A2*** uses binarized counters in the SDM contents matrix and the counter sums are thresholded to form the recalled vector which is compared to the vectors in the item memory using the Hamming distance to select the closest match. The computation of the match Hamming distribution for variant ***A2*** is split into two methods. Method 1 is used if only one row the SDM contents matrix is selected when storing or recalling a vector (that is, m_a equals one). Method 2 is used if more than one row is selected ($m_a > 1$). The code implementing these are in file `binarized_sdm_analytical.py` in class `Binarized_sdm_analytical` (Method 1) and class `Bsa_sample` (Method 2).

1.3.1 Method 1 ($m_a = 1$)

If $m_a = 1$ the match Hamming distribution is calculated as follows. Since m_a is one, there is only one target row (row in the memory that is activated by the address associated with the target data vector). Since there are m rows and one target row, the probability of a random address overlapping the target row is $1/m$. If k vectors are stored in the memory (one target and $k - 1$ other vectors) then the number of possible

overlaps onto the target row ranges from 0 to $k-1$. For a specific case, let j be the number of overlaps onto the target row (j ranges from 0 to $k-1$).

Two arrays are created, both of length $k-1$, each element of which contains a value associated with its index (j) number of overlaps. The first array, `prob_overlap`, contain the probability of each number of overlaps onto the target row. It is set using the binomial distribution by: `prob_overlap[`j`]`$=$ $\mathrm{B.pmf}(j, k-1, 1/m)$, which stands for the binomial distribution calculation for the probability of selecting the target row j times when storing $k-1$ vectors when the probability of selecting the target row when storing one vector is $1/m$. The second array, `delt_overlap`, contains, for each of the possible number of overlaps (j), the probability that the thresholded sum of the counters formed by j overlaps will be in error (not match the corresponding bit in the target data vector). If j is even, the probability of this error is the probability that fewer than $(j/2)$ of the overlap values match the value stored for the target. If j is odd, the probability is the same as for $j+1$ overlaps (result of adding a random vector).[2] To facilitate calculating the `delt_overlap` array for both even and odd j, we define a function, $\mathrm{odd\_up}(x) = x$ if x is even otherwise $x+1$, which converts odd integers to the next larger even integer, but leaves even integers unchanged. With this function, the `delt_overlap` array is created by: `delt_overlap[`j`]`$=$ $\mathrm{B.cdf}(\mathrm{odd\_up}(j)/2-1, \mathrm{odd\_up}(j), 0.5)$, which is the cumulative distribution function (CDF) for the binomial distribution for the probability of $\mathrm{odd\_up}(j)/2-1$ or fewer values matching the target out of $\mathrm{odd\_up}(j)$ values, where the probability of a non-matching value ($+1$ or -1) is 0.5.

After arrays `prob_overlap` and `delt_overlap` are created, the match Hamming distribution is calculated using: $p(h) = \sum_j$`prob_overlap[`j`]` $\mathrm{B.pmf}(h,\ n_c,$ `delt_overlap`$[j])$, where h is a specific Hamming distance (h ranges from 0 to n_c) and the summation is over all possible overlaps (all j) and $\mathrm{B.pmf}(h,\ n_c,$ `delt_overlap[`j`]`$)$ is the probability mass function of the binomial distribution for probability of h successes in n_c trials where the probability of success in each trial is `delt_overlap[j]`. This probability mass function is then used in Equation (5) for $p(H(\mathbf{r}, \mathbf{I}_m) = h)$ (the match Hamming distribution).

1.3.2 Method 2 ($m_a > 1$)

We were unable to develop a method to directly calculate the match Hamming distribution for variant ***A2*** if $m_a > 1$. The method described here generates an estimate for the match Hamming distribution using random sampling and also an empirically derived "correction factor."

Since variant ***A2*** uses binarized counters $\{+1, -1\}$ and m_a counters are added together to form the sums which are threshold to form the recalled vector, only an odd m_a should be used, because if m_a is even, some sums could be zero and a zero sum provides no information about the target bit. So, the method described here assumes that m_a is odd. To ensure that m_a is odd, when computing m_a for memory variant ***A2***, the rounding function in Equation (1) in the main text rounds to the nearest odd integer.

If $m_a > 1$ and odd, the match Hamming distribution is estimated as follows. Since the same row in the SDM contents matrix is never selected more than once by the same address vector, if there are $k-1$ vectors other than the target, the number of possible overlaps onto a single row ranges from 0 to $k-1$ (the same as with Method 1). Since there are m rows, and storing a vector selects m_a rows the

[2] This is because if j is odd the total number of vectors stored (including the target) is $j+1$ which is even, so the counter sum will be even and can be zero. A counter sum of zero has an error probability of .5 which is the same error probability found by adding a random vector (random +1 or -1) to the zero sum. If the sum is not zero (that is the sum is ≤ -2 or ≥ 2) adding a random vector (+1 or -1) will not change the probability because the sum will be on the same side of zero as before.

probability of overlap onto a row from storing one vector is m_a/m. Similar to what is done for Method 1, j represents the number of overlaps onto a target row (ranges from 0 to $k-1$) and two arrays are created: `prob_overlap[`j`]`$=$ B.pmf$(j, k-1, m_a/m)$ contains the probability of j number of overlaps and `delt_overlap[`j`]` contains the probability that the binarized counter will be in error (not match the target bit value) when there are j overlaps. `delt_overlap` is calculated as described in Method 1 above.

In order for the sums of the binarized counters to match their bit in the target data vector, a majority of the counters must be on the right side of zero when they are binarized. In order to facilitate calculating the probability of this occurring, a list is created (called `maski`) which contains all possible combinations of the m_a counters which will result in a "majority vote" that gives correct match. For example, if m_a is three, the majority vote requires at least two of the three binarized counters to be correct to have a correct match. If the counters are identified by a zero-based index, the list would contain tuples: (0, 1), (1, 2), (0, 2), (0, 1, 2); where the first three pairs are all the two-vote majority combinations and the last item is the three vote majority combination. Once this list is determined, the following procedure is used to build up an estimate of the match Hamming distribution. First, an array (named `hdist`) of length n_c+1 (where n_c is the width of vectors in the SDM) is created and initialized to zeros. It will store a histogram of error probabilities for each Hamming distance (Hamming distances ranges from 0 to n_c). Then the following steps are performed repeatedly to build up values in the `hdist` array. First, m_a random samples of overlap numbers are selected from the range of possible overlaps (0 to $k-1$). These random samples are selected according to the probabilities for each overlap in the `prob_overlap` array. This simulates a target vector selecting m_a rows in the memory and provides the number of overlaps onto each of the selected rows.[3]

Once the m_a random samples of number of overlaps are selected, the probability of error in the binarized counters due to each of the number of overlaps is found from the `delt_overlap[j]` array and stored an array of length m_a, named `er`. So array `er`$[i]$ contains the probability that binarized counter i does not match the target bit, where i is the counter number (same number as in the tuple components in the `maski` array), ranging from 0 to m_a-1. Likewise an array `pc` is created with has the probability that each counter is correct (matches the target bit). It is set by `pc[`i`]`$=1-$`er[`i`]`. These arrays are then used to calculate the probability of a correct majority vote by adding the probability of each of the majority vote combinations in the `maski` list. The probability of each majority vote combination (that is each tuple in the `maski` list) is the product of: `pc[`i`]` if the counter i is in the tuple, or `er[`i`]` if counter i is not in the tuple. For example, if $m_a=3$, the probability that the majority vote will be correct is: `pvote=(pc[0]pc[1]er[2])+(er[0]pc[1]pc[2])+(pc[0]er[1]pc[2])+(pc[0]pc[1]pc[2])`. The first three term corresponds to the two-vote majorities in the `maski` list (two of three counters being correct when binarized), the last terms correspond to all three counters being correct. Once the probability of the "vote" (`pvote`) being correct is found, the probability of an error (`delta`) is $1-$`pvote`. This error is used to calculate the probability of each of the possible Hamming distances by $p(h)$ = B.pmf$(h, n_c,$`delta`$)$, where h is the Hamming distance (ranges from 0 to n_c) and B.pmf$(h, n_c,$`delta`$)$ is the probability mass function of the binomial distribution for probability of h successes in n_c trials where the probability of success in each trial is `delta`. The $p(h)$ values are added element-wise to the `hist` array during each iteration. The number of iterations was $100{,}000$. After all the iterations are complete, the `hist` array is normalized so that it is a probability mass function. The

[3] The random sampling could be done with replacement or without replacement. With replacement allows simulating sampling different rows that have the same overlap, but it also allows simulating selecting the same row more than once which does not occur in the SDM. Sampling without replacement ensures simulating selecting different rows, but does not allow simulating selecting different rows that have the same number of overlaps. In our experiments, sampling without replacement—which was used in this study—generates an estimate for the error rate in the final match to item memory that is slightly smaller (less than 2 percent) than sampling with replacement.

normalized `hist` array is then used as the match Hamming distribution to calculate the error rate using Equation (5).

The error rates calculated using the generated `hist` array and Equation (5) are less than the true error rate because the method does not take into account the correlation in overlaps caused by address vectors selecting more than one target row (due to weights greater than 1). However, it was discovered that the difference between the calculated and empirical error when the error was plotted on a log scale, seemed to be a constant. This allows multiplying the calculated error by a correction factor to generate an error estimate that is closer to the empirical error. The correction factor depends on m_a, and for the different values of m_a are: 3 (1.155), 5 (1.921), 7 (3.452). The predicted error rate was formed by multiplying the calculated error by the appropriate correction factor.

1.4 Variant *A3*

The method used to predict the error rate for variant ***A1*** was also used to predict the error rate for variant ***A3*** because it was found empirically that the error rate for ***A1*** and ***A3*** were very close when they have the same number of rows in the contents matrix (m).

1.5 Variant *A4*

The code for associative memory ***A4*** is in file `sdm_ae.py` class `Sdm_error_analytical` and called with parameter `match_method` equal to the string "`both`" or "`dot`". The methods described in Step 1 of variant ***A1*** (algorithm given in Section 1.2.1.2) are used to find the overlap configurations and probabilities (q^g and p^g). For each overlap configuration (each entry in q^g), both a match and distractor distribution are calculated (as described below), and used in Equation (6) in the main text to calculate the probability of correct recall from item memory given that particular overlap configuration. The computed probabilities of correct recall for each overlap configuration are then combined using a weighted sum according to the probability of each overlap configuration given in p^g).[4]

The calculation of the match distribution given a specific overlap configuration (q^g_v) is done as follows. First, the the method described in Section 1.2.2 is used to calculate all the possible sums and the probability of those sums due to the overlaps (but not including the target vector). These are respectively stored in arrays named `cw` (sums) and `cp` (probabilities). Then the contribution of the target vector are included. There are two cases: a $+1$ bit in the target vector increases the sums (`cw`) by m_a, and a -1 bit in the target vector reduces the sums by m_a. This creates two sets of sums and corresponding probabilities; the probabilities are set to the original probabilities (`cp`) divided by 2 since each case (target bit $+1$ or -1) are equally likely. The result of this are the possible counter sums and probability of each sum when recalling the target vector with the particular overlap configuration. Next, the sums for the target bit -1 case are multiplied by -1 which is what would occur if they are multiplied by a bit in the matching item memory vector. The result of this is stored in array `negative_target_sump`. The sums in the +1 target bit case would be multiplied by +1, so they are unchanged; they are stored in array `positive_target_sump`. Then the two groups of sums multiplied by the target bit (`positive_target_sump` and `negative_target_sump`) are combined by concatenating the two arrays and removing any duplicates by finding the sum of the probabilities of the duplicate values and replacing duplicates by one entry in both arrays (the sum array

[4] The reason that a match and distractor distribution is calculated for each overlap configuration (unlike with the other memory variants where a single match and distractor distribution was calculated for all of the overlap configurations) is that for variant ***A4***, the match and distractor distributions depend on the overlap configuration. For example, if there are many overlaps onto the target rows, the range of possible values of the counter sums will be larger than if there are fewer overlaps. A larger range of counter sums will increase the range of possible values of the dot product to both a matching vector and a distractor vector, so the error rate must be calculated using distributions for the match and distractor that are are created by the same overlap configuration.

and the probability array). The end result are arrays (called `match_p`—containing the probabilities, and `match_w`—containing the sums) for the recall of *one bit* of a vector matching the target and multiplying it by that bit of the target in the item memory. All the sums and probabilities constitute a discrete probability distribution with mean `match_mean1`= ⟨`match_p`, `match_w`⟩, (where $\langle \cdot, \cdot \rangle$ denotes the dot product), and variance `match_var1`= ⟨`match_p` , (`match_w`−`match_mean1`)^2⟩ where the subtraction and squaring are done component-wise. These values (`match_mean1` and `match_var1`) are the mean and variance for the value resulting from recalling one bit and multiplying it by the corresponding bit of the matching vector in item memory. The probability distribution for the dot product of all recalled values multiplied by the corresponding bit in the matching vector in item memory (which is the match distribution) is found using the central limit theorem since the dot product is the sum of n_c independent random variables. The mean of the sum is `match_mean_dot`=`match_mean1` n_c, and the variance of the sum is `match_var_dot`= `match_var1` n_c. This completes the computation of the match distribution.

The distractor distribution is calculated in a similar way, except that probability and sums (named, respectively, `distractor_p` and `distractor_w`) are calculated for the recall of one bit of a vector and multiplying it by a random bit, that is, a bit in the item memory that may or may not match the target bit with equal probability. First, the values found by multiplying the possible counter sums by a non-matching bit in item memory are found by inverting the values in the `positive_target_sump` and `negative_target_sump` arrays, and the results are combined into a single array with the probability of each value (before combining) set to 0.25 of the original probability (`cp`) since each case ($+1$ target bit and mismatch to -1 bit in item memory bit; and -1 target bit mismatch to $+1$ bit in item memory) has a 1/4 chance of occurring. The resulting combined arrays, called `no_match_p`, `no_match_w` are then combined with the values for the case of matching to the target (`match_p` and `match_w`) with the probability of the `match_p` divided by 2 since, for the distractor, the probability of a bit matching the bit in item memory is 0.5. The result are two arrays: `distractor_p` and `distractor_w` which constitute a discrete probability distribution for the value found by multiplying a sum formed during recall by a random bit (-1 or $+1$). The mean `distractor_mean1` = ⟨`distractor_p`, `distractor_w`⟩, and variance `distractor_var1`= ⟨`distractor_p`, (`distractor_w`−`distractor_mean1`)^2⟩). These values (`distractor_mean1` and `distractor_var1`) are the mean and variance for the value resulting from recalling one bit and multiplying it by a random bit in item memory. The probability distribution for the dot product of all recalled values multiplied by random bits (a distractor vector in item memory) is the distractor distribution. Similar to the case for the match distribution, it is found using the central limit theorem. The mean is `distractor_mean_dot`=`distractor_mean1` n_c, and the variance is `distractor_var_dot`= `distractor_var1` n_c. This completes the computation of the distractor distribution.

Once the match and distractor distributions are calculated, they are used as described at the top of this section to calculate the probability of correct recall for the particular overlap configuration (q_v^g) used to set up the `cw` and `cp` arrays. The probabilities of correct recall are multiplied by the corresponding probabilities of the overlap in the p^g array and summed to get the overall probability of correct recall. The probability of an incorrect recall is calculated by subtracting the probability of correct recall from 1.
